# Non-pharmacological interventions for smoking cessation: analysis of systematic reviews and meta-analyses

**DOI:** 10.1186/s12916-023-03087-z

**Published:** 2023-09-29

**Authors:** Tao Nian, Kangle Guo, Wendi Liu, Xinxin Deng, Xiaoye Hu, Meng Xu, Fenfen E, Ziyi Wang, Guihang Song, Kehu Yang, Xiuxia Li, Wenru Shang

**Affiliations:** 1 Evidence Based Social Science Research Center/Health Technology Assessment Center, School of Public Health, Lanzhou University, 199 Donggang West Road, Lanzhou, 730000 People’s Republic of China; 2 Key Laboratory of Evidence Based Medicine and Knowledge Translation of Gansu Province, Lanzhou, 730000 People’s Republic of China; 3 Gansu Provincial Medical Security Bureau, Lanzhou, 730000 People’s Republic of China; 4 Vidence Based Medicine Center, School of Basic Medical Sciences, Lanzhou University, Lanzhou, 730000 People’s Republic of China; 5 Collaborative Innovation Center of First Hospital, Lanzhou University, Lanzhou, 730000 People’s Republic of China

**Keywords:** Non-pharmacological approaches, Smoking cessation, Healthy, Evidence map, Systematic reviews/meta-analyses

## Abstract

**Background:**

Although non-pharmacological smoking cessation measures have been widely used among smokers, current research evidence on the effects of smoking cessation is inconsistent and of mixed quality. Moreover, there is a lack of comprehensive evidence synthesis. This study seeks to systematically identify, describe, and evaluate the available evidence for non-pharmacological interventions in smoking populations through evidence mapping (EM), and to search for best-practice smoking cessation programs.

**Methods:**

A comprehensive search for relevant studies published from the establishment of the library to January 8, 2023, was conducted in PubMed, Web of Science, Embase, the Cochrane Library, CNKI, CBM, Wan Fang, and VIP. Two authors independently assessed eligibility and extracted data. The PRISMA statement and AMSTAR 2 tool were used to evaluate the report quality and methodology quality of systematic reviews/meta-analyses (SRs/MAs), respectively. Bubble plots were utilized to display information, such as the study population, intervention type, evidence quality, and original study sample size.

**Results:**

A total of 145 SRs/MAs regarding non-pharmacological interventions for smoking cessation were investigated, with 20 types of interventions identified. The most commonly used interventions were cognitive behaviour education (*n* = 32, 22.07%), professional counselling (*n* = 20, 13.79%), and non-nicotine electronic cigarettes (e-cigarettes) (*n* = 13, 8.97%). Among them, counselling and behavioural support can improve smoking cessation rates, but the effect varies depending on the characteristics of the support provided. These findings are consistent with previous SRs/MAs. The general population (*n* = 108, 74.48%) was the main cohort included in the SRs/MAs. The total score of PRISMA for the quality of the reports ranged from 8 to 27, and 13 studies (8.97%) were rated as high confidence, and nine studies (6.21%) as moderate confidence, in the AMSTAR 2 confidence rating.

**Conclusions:**

The abstinence effect of cognitive behaviour education and money incentive intervention has advantages, and non-nicotine e-cigarettes appear to help some smokers transition to less harmful replacement tools. However, the methodological shortcomings of SRs/MAs should be considered. Therefore, to better guide future practice in the field of non-pharmacological smoking cessation, it is essential to improve the methodological quality of SRs and carry out high-quality randomized controlled trials (RCTs).

**Supplementary Information:**

The online version contains supplementary material available at 10.1186/s12916-023-03087-z.

## Background

At present, tobacco use remains a preventable factor in the occurrence and development of non-communicable diseases, including cardiovascular and respiratory diseases and cancer, and a leading cause of death. In recent years, there has been a relative decline in tobacco use among persons aged 15 years and older, and at the global level, countries are on track to achieve a 22% relative reduction in tobacco use by 2025 [1]. However, despite a steady decline in the number of smokers worldwide, tobacco still kills more than seven million people every year [1]. Smoking has become an increasingly prominent public health problem. Some studies have shown that quitting reduces the risk of major chronic diseases in smokers and can also slow the progression of chronic obstructive pulmonary disease and cancer and extend life expectancy [2, 3]. Helping smokers quit is considered to be the most effective way to reduce the health burden in the short to medium term, while seeking best practice smoking cessation programs would be a cost-effective option, to some extent reducing the heavy economic burden caused by smoking globally [4, 5].

Rigotti et al. classified smoking cessation interventions into psychological and behavioural interventions, drug treatment, and other interventions [6]. Among them, the significance of non-pharmacological smoking cessation has become increasingly evident. Siu et al. concluded that behavioural interventions alone, such as in-person behavioural support and counselling, telephone counselling, and self-help materials, can significantly improve the success rate of tobacco cessation [7]. Ussher et al. demonstrated that abstinence rates were significantly higher in the physically active group than in the control group, especially at the end of the exercise, showing significant benefits [8]. This study only included non-pharmacological intervention research. In addition, we also added an electronic cigarette (e-cigarette) (no nicotine, treatments with nicotine components are classified as drug therapy) intervention [6]. Although non-nicotine e-cigarettes have not been approved as a smoking cessation agent by the Center for Drug Evaluation and Research (CDER) of the US Food and Drug Administration (FDA), they have been promoted for smoking cessation and multiple studies have been published. Meanwhile, analysing the effects of non-nicotine e-cigarettes on withdrawal adds to the range of non-pharmacological smoking cessation studies.

Evidence mapping(EM) is a new comprehensive evidence research method that systematically collects existing evidence in relevant research fields, conducts comprehensive analysis and scientific evaluation, and integrates, condenses, and concisely and intuitively presents the research status, existing problems, development direction, and evidence gap [9, 10]. Unlike umbrella reviews/systematic reviews, which typically involve narrow research questions, the EM describes the volume, design, and characteristics of studies in broad subject areas, and their breadth helps identify research hotspots and evidence gaps while identifying best practice plans [11–13]. Meanwhile, the EM should be included in the literature on high-quality research design. The strength of evidence from SRs/MAs is generally superior to that of single original studies, which is an important basis for the gold standard and practice guidelines for efficacy evaluation [14].

This study, which we plan to include in SRs/MAs, aims to systematically identify, describe, and evaluate available evidence for non-pharmacological interventions in smoking populations using an evidence atlas approach and to identify best practice smoking cessation programs, and research hotspots. To analyse trends in the risk of bias in the included SRs/MAs, we assessed the current state of knowledge and identified evidence gaps for further work.

## Methods

The present study was performed according to the guidelines of Preferred Reporting Items for Overviews of Systematic Reviews including a harms checklist (PRIO-harms) [15]. This evidence map was registered at the OSF Registries (Registration DOI: https://doi.org/10.17605/OSF.IO/R4BZC).

### Data sources and search strategy

In this study, SRs/MAs of smoking cessation studies were comprehensively searched between January 1, 1951, and December 31, 2022 in databases Medline, Web of Science, Embase, Cochrane Library, Cumulative Index to Nursing and Allied Health Literature (CINAHL), PsycINFO, China National Knowledge Infrastructure (CNKI), China Biology Medicine (CBM), Wan Fang, and VIP Database for Chinese Technical Periodicals (VIP). The search keywords included the following: (smok OR cigarette OR tobacco OR nicotine OR cessation OR quit OR Abstinence OR Stop) AND (systematic review OR Overview OR meta-analysis OR meta analyses). The complete search strategies are described in Additional File 1: Table S1 [2–10]. The most recent search was conducted on January 8, 2023, which was a catch-up search.

We also searched the Cochrane Tobacco Addiction Group Specialized Register, checked the list of references for eligible studies by hand-searching at the time of full-text reading, and consulted experts in the field to identify any relevant forthcoming or unpublished studies.

### Eligibility criteria

Based on the principle of Participants, Interventions, Control, Outcomes, and Study Designs (PICOS), we developed the inclusion and exclusion criteria. The following inclusion criteria were applied. First, we included the study population according to the definition of smoking population by the World Health Organization (WHO) International Classification of Diseases 11 (ICD-11), which is not limited by age, sex, and occupation [16]. Second, we deemed the following intervention strategy eligible for inclusion: (a) psychology and behavioural intervention (e.g., cognitive behavioural education, exercise); (b) non-nicotine e-cigarettes; and (c) other interventions (e.g., acupuncture, meditation). Eligible controls were blank, usual care, or other interventions other than those described above. Third, the outcome was to assess the effectiveness or adverse events of non-pharmacological therapy for smoking cessation. Fourth, the included study design was SRs/MAs.

The following studies were excluded: (a) smoking cessation studies with pharmacotherapy-related interventions; (b) nicotine-containing e-cigarettes; (c) no smoking cessation effect was reported in the study outcomes; (d) case reports, review articles, protocols, letters, abstracts, comments, and studies that did not report data; and (e) duplicate reports of the same study.

### Data extraction and management

All the retrieved articles were imported into EndNote X 9.0 software. After excluding duplicate publications, two authors (T.N. and KL.G.) independently screened and extracted data according to the inclusion and exclusion criteria. Disagreements were resolved through a discussion or by consulting a third member (M.X.) with vast experience in the field [17]. After the retrieved literature was deduplicated by EndnoteX9 software, the two authors first screened the studies that might meet the criteria by reading the titles and abstracts according to the inclusion and exclusion criteria and carried out full-text screening of the uncertain studies to determine the final inclusion of all the studies that met the criteria. Data extraction tables were designed using Microsoft Excel 2019 software, and the following information was extracted: first author name, publication year, country, number of RCTs included, interventions, study population, research design, and outcomes of each included study.

### Quality assessment

Preferred Reporting Items for Systematic Reviews and Meta-Analyses (PRISMA) statement and A Measurement Tool to Assess Systematic Review 2 (AMSTAR 2) tool were used to evaluate the reporting quality and methodological quality included in SRs/MAs respectively [18, 19]. It was included independently by two researchers (T.N. and KL.G.), and different opinions were resolved by a third researcher (M.X.).

The PRISMA statement consists of 7 parts and 27 items. Each item is judged according to whether the author reports it or not. A full report is worth one point, a partial report is worth 0.5 points, and no report is worth 0 points. The PRISMA criterion score ≤ 15 was considered to be relatively severe information deficiency, 15–21 was considered to be somewhat defective, and 21–27 was considered to be relatively complete.

AMSTAR 2 considers items 2, 4, 7, 9, 11, 13, and 15 as critical items affecting the preparation and validity of the system evaluation, and the remaining items as non-critical items. A total of 16 items are included, and different items can be judged as “Yes”, “Partial yes”, “No” and “Not applicable”. Finally, the quality levels of high (no or one non-critical area is defective), medium (defect in more than one non-critical area), low (one critical area with or without a non-critical area), and very low (more than one critical area with or without defects in non-critical areas) are calculated. The quality assessment process is conducted online, and the overall quality of the study (“Critical low”, “Low”, “Moderate” and “High”) is automatically generated after the completion of the assessment results [20].

### Data synthesis and statistical analysis

Microsoft Excel 2019 was used to extract and manage the data. The frequency and percentage of descriptive statistics were used to analyse the data and generate numbers. A bar chart was utilized to show the reporting quality and methodological quality results of the included SRs. We used a bubble plot to bring the included SRs/MAs together and display information on four dimensions, including the smoking cessation effect of SR inclusion, quality of evidence, population, and intervention [21]. Details are as follows: (a) authors’ conclusions (“Effective”, “Likely effective”, “Uncertain” and “Ineffective”) on the *x*-axis; (b) score from AMSTAR 2 assessment on the *y*-axis; (c) each bubble represents one SR, the color represents the population, and the size represents the number of people; and (d) the letters on the bubbles represent interventions.

For descriptive purposes, we categorized conclusions reported by authors for each PICO question, into four categories: “Effective”, “Likely effective”, “Uncertain” and “Ineffective”, as in the categorization performed in previous EM [22]. See Table 1.

**Table 1:** Classification of the conclusions according to results reported by authors

Classification	Definition
Effective	The conclusions reported a clear beneficial effect without major concerns regarding the supporting evidence
Likely effective	The conclusions did not claim for firm beneficial effect despite the reported positive treatment effect
Uncertain	The direction of results differed within reviews due to conflicting results or limitations of individual studies
Ineffective	The conclusions provided evidence of no difference between the intervention and comparator

## Results

### Literature selection

**Fig. 1:**
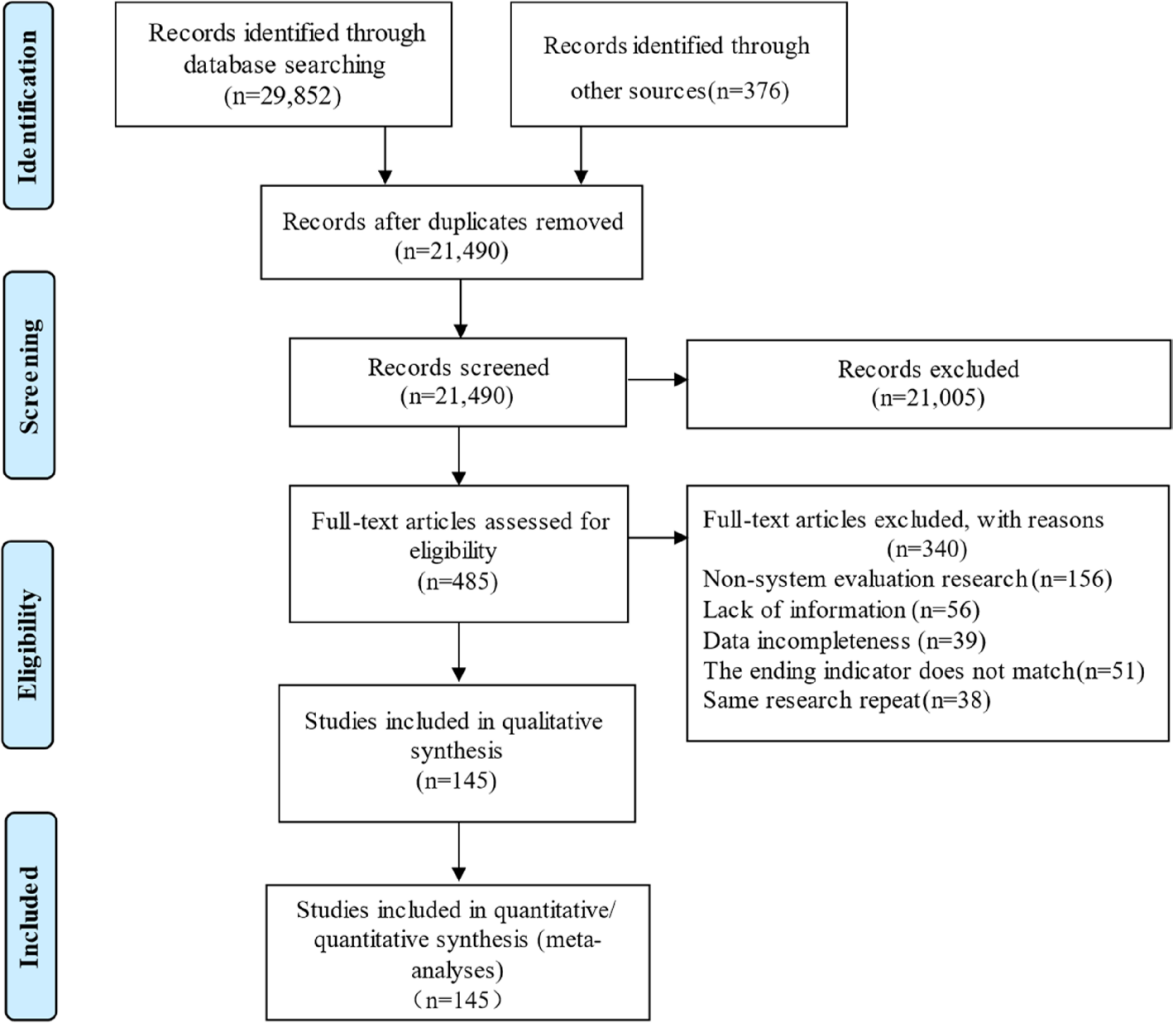
Study selection flowchart

We initially identified 30,228 relevant articles according to the search strategy. Of these, 8738 studies were excluded because of duplication, 21,490 studies were screened by the titles and abstracts, and 485 studies were assessed through the full texts. Finally, 145 SRs/MAs were included in this study [23–167]. The literature screening procedure is shown in Fig. 1.

### Study characteristics

Among 145 SRs/MAs, a quantitative synthesis (meta-analysis) accounted for 71.72%. The years of publication of studies were distributed from 1996 to 2022, and a majority of the studies were published after 2015. The years with 10 or more articles were 2017 (*n* = 14, 9.66%), 2019 (*n* = 17, 11.72%), 2021 (*n* = 10, 6.90%), and 2022 (*n* = 15, 10.34%). According to the country of origin of the first author, there are 12 countries with two or more published studies, among which the top three countries are the United Kingdom (UK) (*n* = 47; 32.41%), the United States (US) (*n* = 35; 24.14%) and Australia (*n* = 19; 13.10%). A total of 2670 individual studies were analysed in the included SRs/MAs, and 93 SRs/MAs (64.14%) included more than ten individual studies. In terms of population characteristics, 108 studies (74.48%) included mixed populations (population characteristics were not divided, mixed with various characteristics of the population), special populations including pregnant women (*n* = 9, 6.21%), acquired immunodeficiency syndrome (AIDS) patients (*n* = 3, 2.07%) and other vulnerable groups (referring to those with weak social power and power and difficult living conditions) (*n* = 8, 5.52%). A total of 20 non-pharmacological smoking cessation methods were included in the study. The commonly used intervention measures were cognitive behaviour education (*n* = 32, 22.07%), professional counselling (*n* = 20, 13.79%), and non-nicotine e-cigarette use (*n* = 13, 8.97%). The details are listed in Table 2.

**Table 2:** Report characteristics of incorporating system evaluation

Project	Category	Number of studies (%)
**Study**	Quantitative synthesis	104(71.72)
	Qualitative synthesis	41(28.28)
Year	2021–2022	25(17.24)
	2016–2020	54(37.24)
	2011–2015	32(22.07)
	2006–2010	20(13.79)
	2000–2005	11(7.59)
	–1999	3(0.69)
**Country**	UK	47(32.41)
	US	35(24.14)
	Australia	19(13.10)
	Canada	8(5.52)
	China	9(6.21)
	Germany	4(2.76)
	South Korea	4(2.76)
	Netherlands	3(2.07)
	India	3(2.07)
	New Zealand	2(1.38)
	Austria	2(1.38)
	Spain	2(1.38)
	Other^a^	6(4.14)
**Population**	General populations	108(74.48)
	Special populations	22(15.17)
	Vulnerable populations	8(5.52)
	Other populations^b^	7(4.83)
**Intervention**	Cognitive behavioural education	32(22.07)
	Professional advice	20(13.79)
	Non-nicotine e-cigarettes	13(8.97)
	Internet interventions	9(6.21)
	Smokeless policy	7(4.83)
	Telephone and SMS counseling	7(4.83)
	Group support	7(4.83)
	Acupuncture	9(6.21)
	Game incentives	4(2.76)
	Motive interview	4(2.76)
	Exercise	10(6.87)
	Mixed psychological intervention	3(2.07)
	Quit smoking Application (App)	4(2.76)
	Money incentives	3(2.07)
	Brain stimulation	4(2.76)
	Self-help programs	3(2.07)
	Meditation	3(2.07)
	Other^c^	3(2.07)

^a^Other countries that published only one study: Denmark, Malaysia, Norway, Thailand, Iran, and Italy

^b^Other populations involved in only one study: cancer patients, coronary heart disease (CHD), elderly, schoolgirls, and patients with cardiovascular disease

^c^Other interventions involving only one study: hypnotherapy, smoking reduction, and aversion therapy

### Reporting quality of the included SRs/MAs

**Fig. 2:**
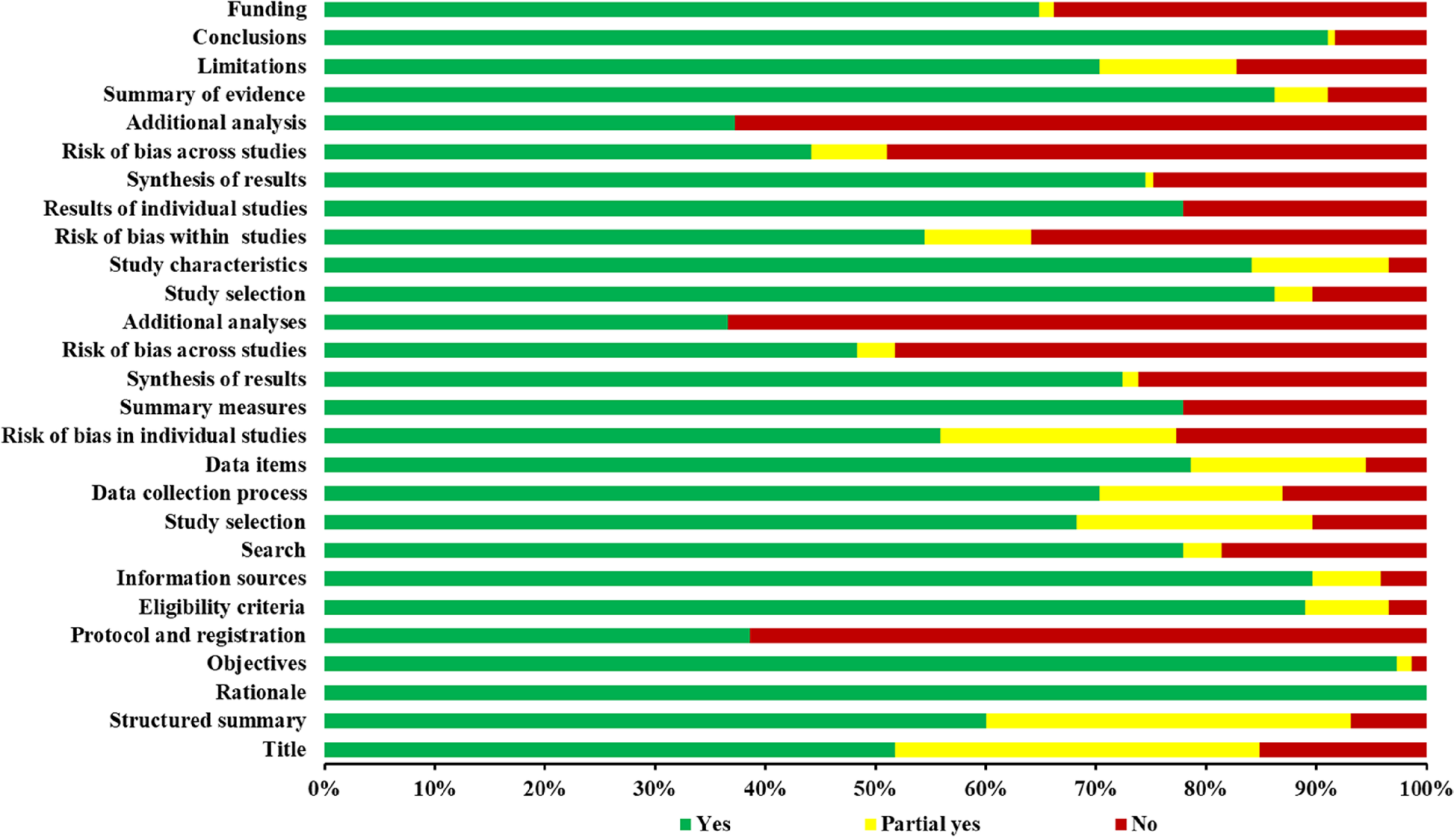
Reporting quality of the included SRs/MAs

The quality evaluation results of the PRISMA report are shown in Fig. 2. For the 27 PRISMA items, the theoretical basis (item 3) and research objective (item 4) were well reported, with more than 97% of the SRs/MAs describing these two items in the background introduction. Eight items had reporting rates of more than 80% (items 3, 4, 6, 7, 17, 18, 24, 26), and only three items were less than 50% (items 5, 16, 23). The total PRISMA score for the quality of the included studies ranged from 10 to 27. There were seven articles with a score of less than or equal to 15, 38 articles with a score of more than 15 and less than or equal to 21, and 59 articles with a score of more than 21 and less than or equal to 27. The PRISMA quality appraisal scores are presented in Additional File 1: Table S2) [11–19].

### Methodological quality of the included SRs/MAs

**Fig. 3:**
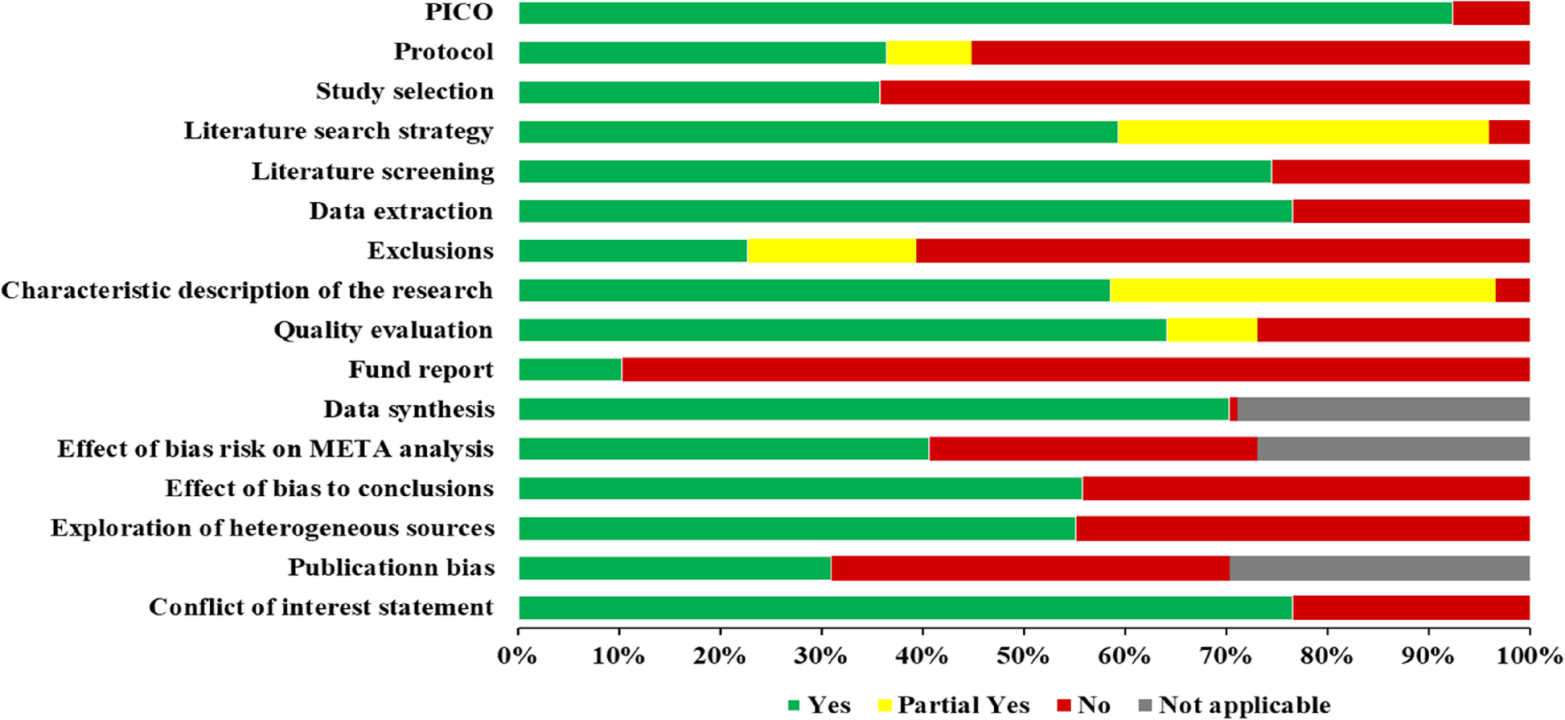
Methodological quality of the included SRs/MAs

The results of the AMSTAR 2 assessment are shown in Fig. 3. For each AMSTAR 2 item, 5 of 16 items were rated as relatively complete, with reporting rates ≥ 70% (items 1, 5, 6, 11, and 16). A total of 53 studies (36.55%) reported the predefined protocol (item 2), 52 studies (35.86%) provided the reason for inclusion (item 3), 86 studies (59.31%) provided the comprehensive search strategy and supplementary search (item 4), 33 studies (22.76%) provided the reason for exclusion (item 7), 85 studies (58.62%) described the basic characteristics of the research (item 8), 93 studies (64.14%) provided the appropriate risk of bias tools for the reviews (item 9), 15 studies (10.34%) reported to research funding sources (item 10), 59 studies (40.69%) assessed the potential effect of the risk of bias of individual studies (item 12), 81 studies (55.86%) accounted for the risk of bias in individual studies when interpreting the results (item 13), and 80 studies (55.17) provided a satisfactory explanation for heterogeneity in the review results (item 14). Publication bias was explained in 45 studies (31.03%) (item 15). For overall methodological quality, 13 studies (8.97%) were rated as high confidence, ten studies (6.90%) were rated as moderate confidence, and 25 studies (17.24%) were rated as low confidence. Ninety-seven studies (66.90%) were assessed as having very low confidence. The AMSTAR 2 quality appraisal scores are presented in Additional File 1: Table S3 [20–26].

### Efficacy outcomes

**Fig. 4:**
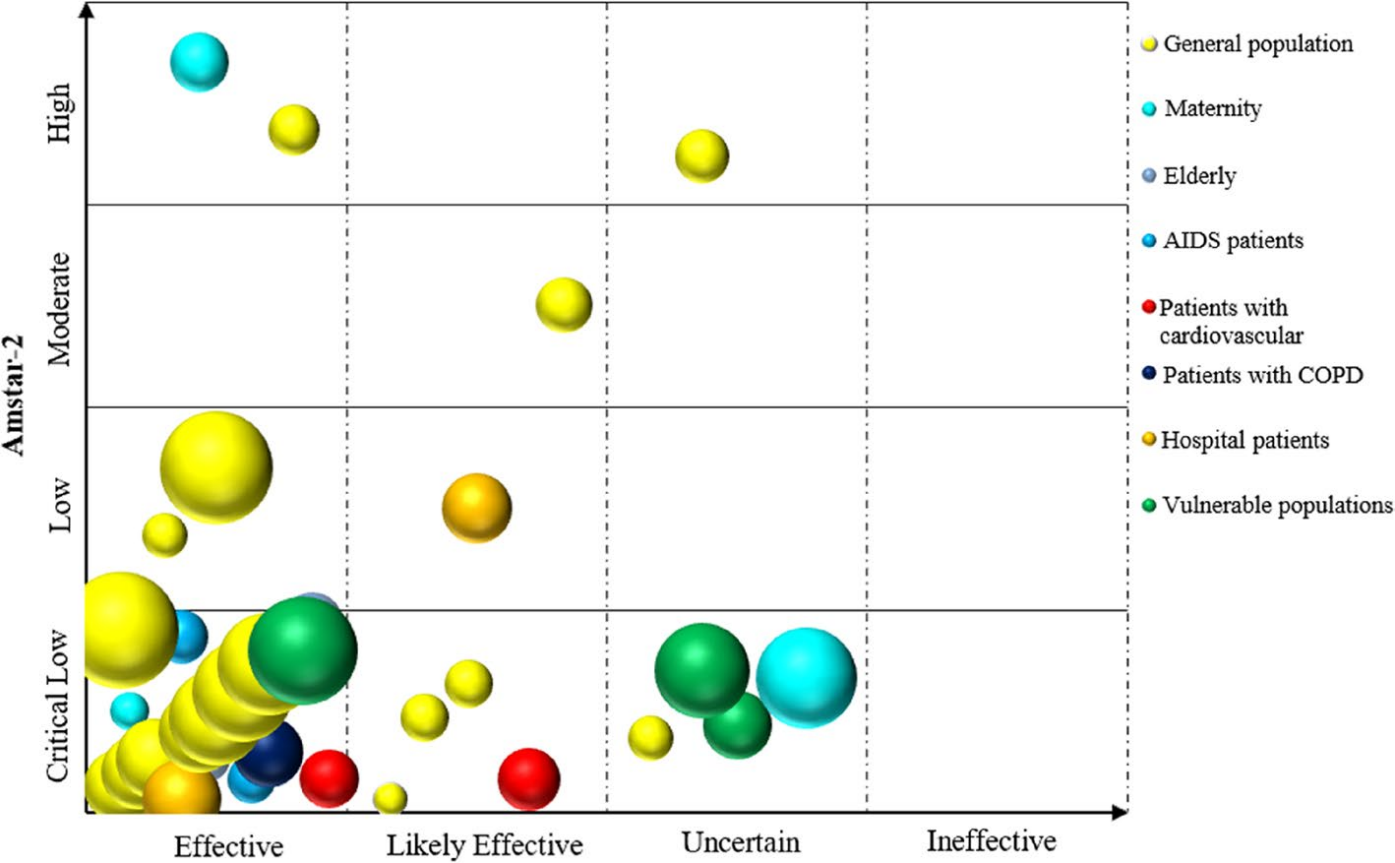
Cognitive behavioural education intervention to quit smoking affects different people

According to the results of the integrated inclusion studies, 32 SRs/MAs have centered around cognitive behavioural education interventions. As shown in Fig. 4, after psychological and behavioural intervention in the general population (*n* = 17), 21 of them were “Effective” outcomes, six were “Likely effective” and five were “Uncertain”. Of these studies, three possessed high to moderate evidence quality, while the remaining 14 featured low to very low quality. Moreover, three research results documented smoking cessation in pregnant women, of which two realized “Effective” effects. Studies aimed at elderly patients, AIDS sufferers, and chronic obstructive pulmonary disease (COPD) patients (*n* = 2, *n* = 2, and *n* = 1, respectively) all delivered “Effective” outcomes, whereas cardiovascular and inpatient cases (*n* = 2 and *n* = 2, respectively) generated one “Effective” and one “Likely effective”.

**Fig. 5:**
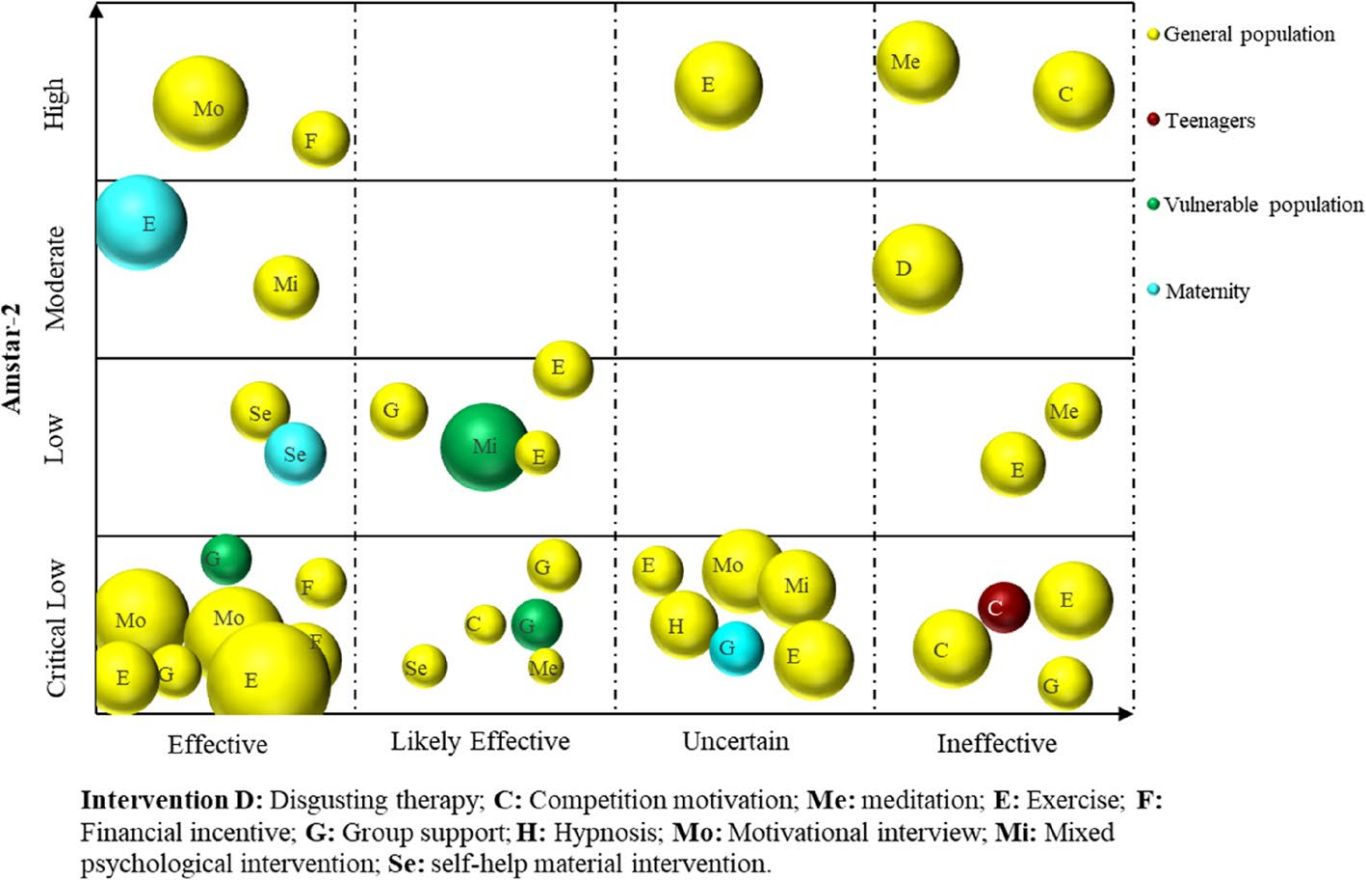
Effect of relevant intervention on smoking cessation in different populations

As depicted in Fig. 5, a total of 39 SRs/MAs were integrated. The results indicated that smoking cessation interventions (including motivational interviews, financial incentives, exercise, mixed psychological interventions, self-help material interventions, and group support) were “Effective” in 14 studies. Of note, vulnerable groups indicated significant effects due to group support and mixed psychological interventions. The smoking cessation effect of pregnant women through exercise and self-help material intervention was effective. Furthermore, nine studies with regard to smoking cessation interventions (including competition motivation, exercise, meditation, group support, mixed psychological intervention, and self-help material intervention) were “Likely effective”. Seven studies noted “Uncertain” outcomes regarding their smoking cessation interventions (involving exercise, group support, hypnosis, motivational interview, and mixed psychological intervention). Finally, nine studies (including disgusting therapy, competition motivation, meditation, exercise, and group support) yielded “Ineffective” results for smoking cessation. As far as methodological quality is concerned, eight studies were classified as being of high to medium quality, while the remaining featured low to very low quality.

**Fig. 6:**
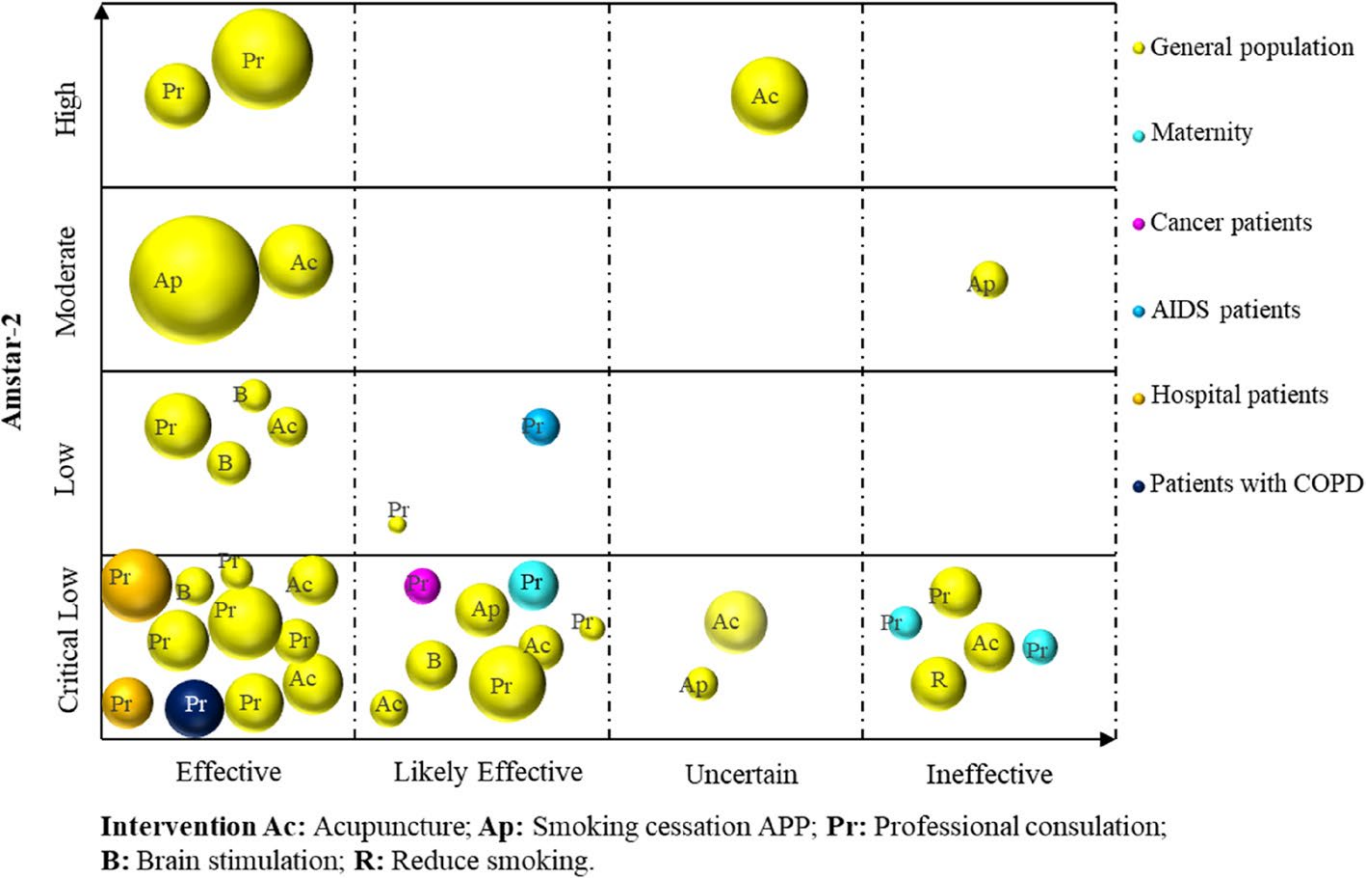
Effect of relevant intervention on smoking cessation in different populations

As demonstrated in Fig. 6, 38 SRs/MAs were incorporated. The results indicated that smoking cessation interventions (including acupuncture, smoking cessation App, professional guidance, and brain stimulation) yielded “Effective” outcomes in 19 studies, of which interventions involving professional consultation with cancer, AIDS, and other hospital patients yielded clear results. Ten studies displayed “Likely effective” results related to smoking cessation interventions (including acupuncture, smoking cessation App, professional consultation, and brain stimulation). Furthermore, three studies about interventions such as acupuncture and smoking cessation Apps were identified as “Uncertain”. Six studies showed that the respective interventions (including acupuncture, smoking cessation App, professional consultation, and smoking reduction) were “Ineffective”. In terms of methodological quality, six had high or medium quality, whereas the remaining had low or very low quality.

**Fig. 7:**
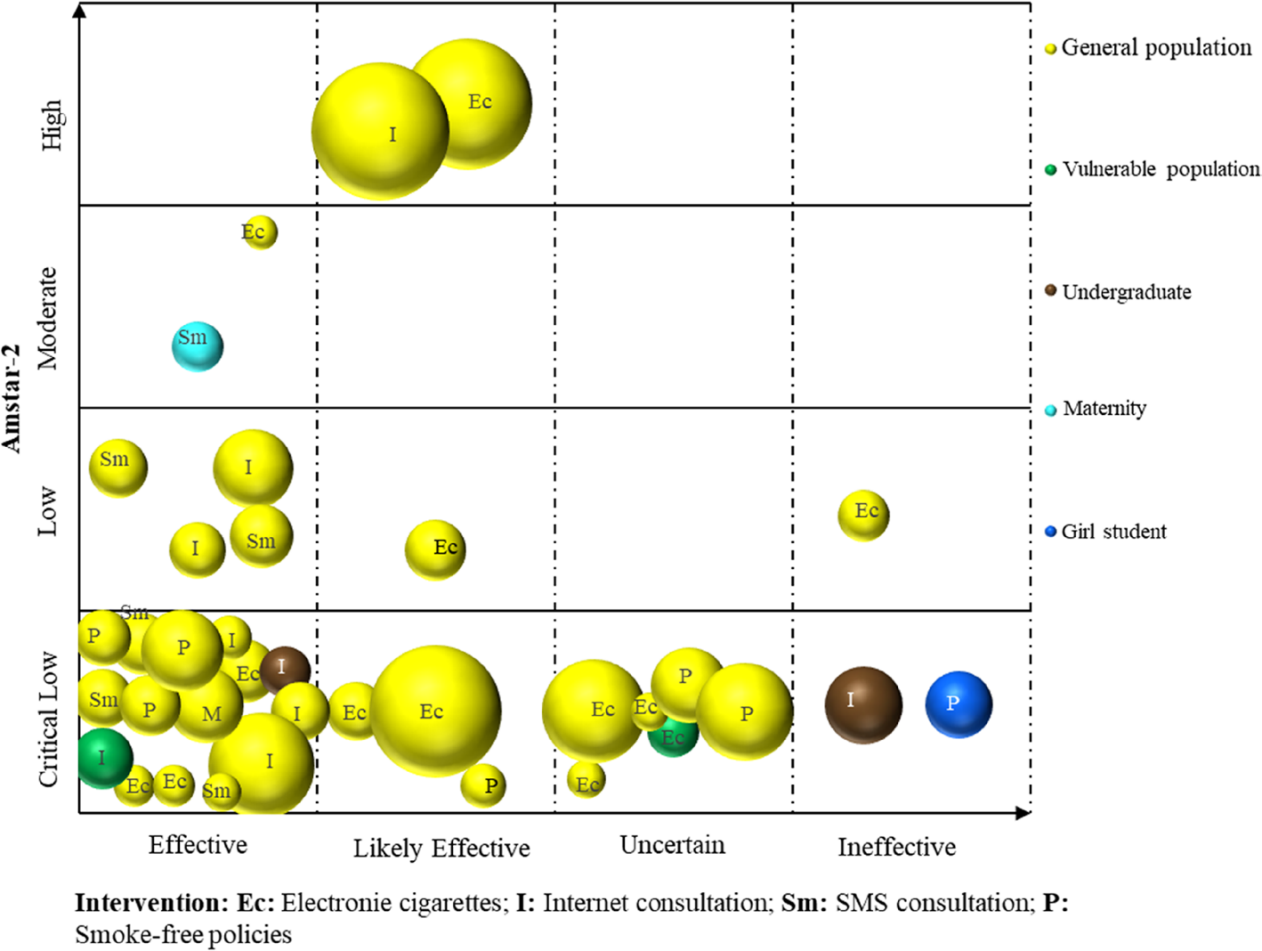
Effect of relevant intervention on smoking cessation in different populations

As demonstrated in Fig. 7, a total of 36 SRs/MAs were included, concluding that smoking cessation interventions (including non-nicotine e-cigarettes, Internet consultation, SMS consultation, and smoke-free policies) effectuated an “Effective” outcome in 21 studies. Six studies revealed that such interventions (again, incorporating non-nicotine e-cigarettes, online consultancy, and smoke-free policies) constituted “Likely effective” results; six rendered an “Uncertain” verdict; and three studies concluded that they were “Ineffective”. Of these studies, four studies featured high to medium methodological quality, while the others were low to very low.

## Discussion

### Summary of the main findings

The EM can amplify the comprehension of a specific field’s direction and trend. This study applied it to render the four-dimensional representation of included SRs/MAs (methodological quality, smoking cessation effects, interventions, and population), compare the variation in smoking cessation effects among different populations and interventions, and delve into treatment effects and study quality. The research publications in the past decade have been at a high level, mainly reviewed and analysed by British and American scholars, and it is a hot research topic in the treatment of persistent smokers. We collected data from 145 SRs/MAs studies distributed between 1996 and 2022, a large number of which reflect the growing therapeutic potential value and interest in non-pharmacotherapy interventions in smoking populations. Of the various interventions observed, results determined that 51.72% of studies regarded them as effective in facilitating persistent smokers quitting, 31.38% were likely to be effective, 14.48% were uncertain, and 12.41% were ineffective.

We ascertained that several abstinence measures, such as cognitive behavioural education, professional counselling advice, and motivational interviews, were efficacious in raising smokers’ cognizance of the connection between smoking and illness through various face-to-face avenues, thereby reducing smoking rates [129, 168, 169]. Cognitive behavioural education can provide the population with a well-developed smoking cessation program, a proper understanding of nicotine addiction, and skills to cope with cravings and negative emotions to maintain abstinence compared to conventional controls. The smoking cessation effect of behavioural interventions shown in our study is consistent with the outcomes of past network meta-analyses [170]. Notably, 88% of the studies on the effect of cognitive behavioural education on withdrawal in the general population were “Effective” or “Likely effective”. The impacts of professional counselling are likewise noteworthy, especially in the short to mid-term, echoing the findings of Lancaster et al. [171]. These observations imply that its efficiency may be mirrored in the readiness and powerful motivation of smokers themselves, compelling them to obtain information regarding smoking cessation through consulting professional doctors [172]. Conversely, Lindson et al. demonstrated that motivational interviews are more suitable for those with low motivation to quit smoking [169]. Moreover, the implementation of motivational interviews is also critical. The effect of motivational interviews conducted by nurses is not significant, and the motivational interviews provided by general practitioners will bring more benefits than those provided by nurses or consultants. This may be because general practitioners and smokers are already familiar with and have established a good personal relationship, and this state is more suitable for this approach [138]. However, this inference is based on a few relatively small studies and must not be exaggerated. Of course, in addition to smoking counselling, smoking cessation rates can be monitored for controllable smoking risk factors. As early as 2005, the Chinese government ratified the WHO Framework Convention on Tobacco Control, which was successfully implemented in major cities such as Shanghai and Beijing, significantly mitigating smoking rates in these areas. However, implementation capacity and supervision fluctuate substantially among provinces and regions (urban and rural), resulting in varying smoking cessation effects [173]. Furthermore, prohibiting tobacco sponsorship and advertising exposure, disallowing sales to minors, escalating taxes and prices, and being informed on the dangers of smoking have collectively contributed to diminishing smoking rates to some degree [174].

With the burgeoning prevalence of the Internet, smoking cessation techniques rooted online have aroused remarkable interest. Most of the relevant literature we searched and included was published in the past 10 years. Originally, interventions primarily entailed network consultations and SMS messaging. In agreement with previous MAs, the evidence indicates that the majority of these modalities demonstrate some degree of abstinence effect on smokers [170]. Notably, active telephone counselling has exhibited efficacy [88]. This bidirectional interactive intervention, such as text messaging and other up-to-date information and communication technologies, allows smokers to acquire smoking cessation information via the web or on the phone and text messages, and through asynchronous and real-time messaging with support networks, in addition to reducing barriers such as cost, location or time/schedule constraints, promoting the implementation of smoking cessation measures [175]. Furthermore, extended communication amplifies user participation in smoking cessation programs, can efficaciously boost the recognition of smoking cessation, and diminish smoking and corporeal and mental dependence on tobacco [176, 177]. Currently, with the emerging trend of smoking cessation Apps, evidence of beneficial effects has been overwhelmingly restricted to follow-up of 6 months or less, yet there is scant proof of long-term abstinence through a smoking cessation App. Do et al. conjecture that web-based and structured text messaging aids may be more likely to increase long-term smoking cessation effects [163].

Non-nicotine e-cigarette interventions are similar to but do not fall under the category of alternative therapy, and aim to maintain smoking cessation habits, using the stimulation of smoking behaviour to reduce withdrawal symptoms when quitting [178, 179]. Batra et al. indicated that nicotine addiction among smokers is a complex behaviour that depends not only on environmental and inherited components but also on psychological features and habits [180]. Non-nicotine e-cigarette intervention maintains the habit of smoking, is safer than cigarettes, and reduces irritability, depression, and withdrawal symptoms of craving [179]. However, in our findings, the smoking cessation effectiveness of non-nicotine e-cigarettes varied according to the characteristics of the population, which is consistent with the results of the review by Hartmann et al. [181]. The use of non-nicotine e-cigarettes has helped reduce the use of paper cigarettes to some extent, but reducing smoking may not increase the time it takes current smokers to quit, and most circumstantial evidence has found that reducing smoking is associated with the likelihood of quitting in the future [109].

We also grouped other non-pharmacological interventions. The results of the investigations into the influence of exercise on abstinence were contradictory and mostly indicated a temporary effect at the end of the exercise [182, 183]. Although exercise does not generally increase the length of time for quitting smoking, it has the potential to offer benefits. Daley et al. uncovered that exercise can aid in lessening the development of many clinical disorders, abating the risk of future disease, and decreasing withdrawal symptoms, such as anxiety and mood swings resulting from giving up smoking [184–186]. The intervention of motivation mechanisms (monetary motivation or competition motivation) is generally arduous to effectuate due to the complexity of the original research design and appraisal. Moreover, confounding factors such as income, gender, and occupation contribute to a high risk of selective bias leading to conflicting research outcomes [42, 187]. Smoke-free policies reduce the prevalence of tobacco use in the population by reducing smokers’ consumption and augmenting attempts to quit, thus increasing the number of successful quitters [72]. However, the potency of smoking cessation is usually undermined by the location in which it is conducted [37]. However, we note that although most of the conclusions extracted from SRs/MAs are classified as “Effective” or “Likely effective”, the evidence for non-pharmacological smoking cessation effects interventions is not entirely the same. The included SRs/MAs claimed inconsistent or even contradictory conclusions about some of the same interventions, such as match motivation, team support, aversion therapy, meditation, and acupuncture. Because the inclusion of SRs is limited, there is insufficient evidence that they are effective forms of treatment. In fact, for the effect of smoking cessation that we reflected in the EM, some studies could not draw firm conclusions despite randomized controlled trials.

Because of the particularity of the population, different populations have different sensitivities to the same interventions. Studies on inpatients, such as AIDS patients, cardiovascular patients, and COPD patients, indicate improved adherence to smoking cessation among those who partake in professional physician counselling and receive cognitive behavioural education from nursing staff [60, 98, 138]. A study performed by Stead et al. revealed that people suffering from co-morbidities have increased levels of anxiety and that advice provided by medical personnel may partially mitigate their apprehensive state [100]. Furthermore, medical providers should strive to establish a good connection with these smokers. Concerning vulnerable groups, evidence suggests that a team-based approach to smoking cessation produces more significant results, likely attributed to the social and psychological support provided in those circumstances and the resulting betterment of mental health [46]. de Kleijn et al. analysed the effect of school-centered intervention combined with mass media intervention by conducting experiments on 12- to 13-year-old female students and the results were significant [188]. The analysis may be that children in this age group, especially girls, are highly influenced by their peers [189].

Although the EM can only provide an overview of a wide range of research areas, the results suggest that there are more valid or potentially valid conclusions than there are uncertain or inefficient ones. However, the quality of the included systematic review studies was mostly low or very low. According to PRISMA, the reporting quality of the included quantitative SRs/MAs has several shortcomings. There were seven SRs with relatively serious insufficient information and 38 SRs with certain defects. The main defects were not clearly stated in the title that the research was a systematic review or meta-analysis; no registered research proposal or report in the paper; failure to describe possible bias in the method part and analysis in the results part; and heterogeneity arising from data consolidation was not analysed. The reported shortcomings of qualitative systematic reviews focused on registration protocols and possible bias in each study. In addition, attention should be given to several limitations related to the quality of the methodology included in the SR, particularly the seven important evaluation areas. Under AMSTAR, 2,97 SRs/MAs were assessed as having very low confidence and 25 were assessed as having low confidence, mainly due to the failure to provide content in the following key evaluation areas, prestudy protocols; no list of excluded studies, and reasons for exclusion. The possibility of publication bias was not adequately investigated after quantitative consolidation, and the effect of publication bias on the results was discussed. In addition, items 3, 10, and 12 also need to be improved. All of the above limitations affect confidence in SR inclusion.

### Evidence gaps and future research directions

The results of the evidence atlas suggest that there may be gaps in non-pharmacological smoking cessation interventions in smokers (1). The methodological quality of the studies was generally low. The quality of research is important for the practice and promotion of intervention measures and scientific research results. The EM results showed that the included SRs/MAs were of low quality, with only 9.23% of the articles rated as high-quality studies. The new study should correct this to some extent. Depending on the quality of the evidence, future reviews should register research protocols in advance and take full account of heterogeneity and publication bias arising from data consolidation. (2) At present, most research on the effectiveness of the intervention of existing contradictions, mainly includes independent intervention, reducing smoking, money motivation, competition motivation, smoke-free policies, team support, mixed psychological intervention, motivated interviews, quit App consulting, exercise, aversion therapy intervention, non-nicotine e-cigarettes, acupuncture intervention, hypnosis, and meditation. This requires further high-quality original studies and SRs/MAs in the future to clarify their effects. (3) As the basis of clinical practice guidelines, systematic evaluation/meta-analysis is extremely important for the practice of intervention. However, the detailed implementation process of intervention, such as intervention time and intensity, is rarely involved in systematic evaluation/meta-analysis, which affects the promotion and implementation of the intervention. (4) Relapse often occurs in the process of quitting smoking, and there are many reasons for relapse. Currently, there is a lack of research, which needs to be further explored by high-quality research in the future.

### Strengths and limitations

This study has several strengths. First, we conducted a comprehensive search from ten databases to identify SRs/MAs associated with non-pharmacological intervention for smoking cessation. Second, we assessed the reporting and methodological quality of the included SRs using PRISMA and AMSTAR 2 tools. Third, the EM, a visualization method, was utilized to present the trends and gaps in the risk of bias of SRs, as well as relationships between evidence outcomes and populations and interventions.

Our study has several limitations. First, this study only included a SRs/MAs, and excluded other study designs (such as randomized controlled trials, cohort studies, and case‒control studies. Second, there were some differences in the clinical trial inclusion criteria of each SR: some included retrospective studies instead of real prospective randomized controlled trials. Third, our results are based only on publications published before January 8, 2023, and need to be updated as new studies emerge. Fourth, the language was restricted to English or Chinese. Literature reviews in other languages were not included, causing a potential language bias.

## Conclusions

In conclusion, the quality of non-pharmacological smoking cessation interventions for smokers is generally low. The same interventions have different effects on smoking cessation in different studies, and even opposite conclusions have been drawn. Future researchers still need to pay attention to differences in the effectiveness of different interventions, intensity and duration, adverse effects of interventions, and methodological quality of studies.

### Supplementary Information

**Additional file 1: Table S1.** Search Strategy. **Table S2.** PRISMA quality appraisal scores. **Table S3.** AMSTAR 2 quality appraisal scores.

## Data Availability

Not applicable.

## Abbreviations

AIDS: Acquired immunodeficiency syndrome
AMSTAR 2: A Measurement Tool to Assess Systematic Review-2
App: Application
CBM: China Biology Medicine
CDER: Center for Drug Evaluation and Research
CHD: Coronary heart disease
CINAHL: Cumulative Index to Nursing and Allied Health Literature
CNKI: China National Knowledge Infrastructure
COPD: Chronic obstructive pulmonary disease
e-cigarettes: Electronic cigarette
EM: Evidence mapping
FDA: Food and Drug Administration
ICD-11: International Classification of Diseases 11
PICOS: Participants, Interventions, Control, Outcomes, and Study Designs
PRIO: Preferred Reporting Items for Overviews
PRISMA: Preferred Reporting Items for Systematic Reviews and Meta-Analyses
RCTs: Randomized controlled trials
SRs/MAs: Systematic Reviews/Meta-Analysis
UK: United Kingdom
US: United States
VIP: VIP Database for Chinese Technical Periodicals
WHO: World Health Organization
