# Supplementary material for: Non-pharmacological interventions for smoking cessation: analysis of systematic reviews and meta-analyses
Source: BMC Med. 2023 Sep 29;21:378. doi: 10.1186/s12916-023-03087-z (PMC10542700; doi:10.1186/s12916-023-03087-z)
Supplement: Supplementary file 1 — Additional file 1: Table S1. Search Strategy. Table S2. PRISMA quality appraisal scores. Table S3. AMSTAR 2 quality appraisal scores. [file 12916_2023_3087_MOESM1_ESM.docx]

**Additional File 1**

**Table S1): Search Strategy**

**Table S2): PRISMA quality appraisal scores**

**Table S3): AMSTAR 2 quality appraisal scores**

**Table S1): Search Strategy**

| **PubMed 4390** |
| --- |
| #1 cigarette smoking [Mesh] OR tobacco [Mesh] OR nicotine [Mesh]  #2 smok* [Title/Abstract] OR cigarette [Title/Abstract] OR tobacco [Title/Abstract] OR nicotine [Title/Abstract]  #3 #1 OR #2  #4 cessation [Title/Abstract] OR cease [Title/Abstract] OR quit*[Title/Abstract] OR abstinence [Title/Abstract] OR abstinent [Title/Abstract] OR stop*[Title/Abstract] OR reject [Title/Abstract] OR reduce [Title/Abstract] OR resist [Title/Abstract] OR resistance [Title/Abstract] OR give up [Title/Abstract] OR withdraw [Title/Abstract]  #5 systematic[Subject] OR meta-analysis[Publication Type] OR meta-analysis as topic[Mesh] OR meta-analysis[Mesh] OR meta analy*[Text Word] OR metanaly*[Text Word] OR metaanaly*[Text Word] OR met analy*[Text Word] OR integrative research[Title/Abstract] OR integrative review*[Title/Abstract] OR integrative overview*[Title/Abstract] OR research integration*[Title/Abstract] OR research overview*[Title/Abstract] OR collaborative review*[Title/Abstract] OR collaborative overview*[Title/Abstract] OR systematic review*[Title/Abstract] OR technology assessment*[Title/Abstract] OR technology overview*[Title/Abstract] OR "Technology Assessment, Biomedical"[Mesh] OR HTA[Title/Abstract] OR HTAs[Title/Abstract] OR comparative efficacy[Title/Abstract] OR comparative effectiveness[Title/Abstract] OR outcomes research[Title/Abstract] OR indirect comparison*[Title/Abstract] OR ((indirect treatment[Title/Abstract] OR mixed-treatment[Title/Abstract]) AND comparison*[Title/Abstract]) OR Embase*[Title/Abstract] OR Cinahl*[Title/Abstract] OR systematic overview*[Title/Abstract] OR methodological overview*[Title/Abstract] OR methodologic overview*[Title/Abstract] OR methodological review*[Title/Abstract] OR methodologic review*[Title/Abstract] OR quantitative review*[Title/Abstract] OR quantitative overview*[Title/Abstract] OR quantitative synthes*[Title/Abstract] OR pooled analy*[Title/Abstract] OR Cochrane[Title/Abstract] OR Medline[Title/Abstract] OR Pubmed[Title/Abstract] OR Medlars[Title/Abstract] OR handsearch*[Title/Abstract] OR hand search*[Title/Abstract] OR meta-regression*[Title/Abstract] OR metaregression*[Title/Abstract] OR data synthes*[Title/Abstract] OR data extraction[Title/Abstract] OR data abstraction*[Title/Abstract] OR mantel haenszel[Title/Abstract] OR peto[Title/Abstract] OR der-simonian[Title/Abstract] OR dersimonian[Title/Abstract] OR fixed effect*[Title/Abstract] OR "Cochrane Database Syst Rev"[Journal] OR "health technology assessment winchester, england"[Journal] OR "Evid Rep Technol Assess (Full Rep)"[Journal] OR "Evid Rep Technol Assess (Summ)"[Journal] OR "Int J Technol Assess Health Care"[Journal] OR "GMS Health Technol Assess"[Journal] OR "Health Technol Assess (Rockv)"[Journal] OR "Health Technol Assess Rep"[Journal].  #3 AND #4 AND #5 |
| **Cochrane 1331** |
| #1 MeSH descriptor: [cigarette smoking]  #2 MeSH descriptor: [tobacco]  #3 MeSH descriptor: [nicotine]  #4 smok* OR cigarette OR tobacco OR nicotine  #5 cessation OR cease OR quit* OR abstinence OR abstinent OR stop* OR reject OR reduce OR resist OR resistance OR give up OR withdraw  #6 systematic OR meta-analysis OR meta-analysis as topic OR meta-analysis OR meta analy* OR metanaly* OR metaanaly* OR met analy* OR integrative research OR integrative review* OR integrative overview* OR research integration* OR research overview* OR collaborative review* OR collaborative overview* OR systematic review* OR technology assessment* OR technology overview* OR "Technology Assessment, Biomedical" OR HTA OR HTAs OR comparative efficacy OR comparative effectiveness OR outcomes research OR indirect comparison* OR ((indirect treatment OR mixed-treatment) AND comparison*) OR Embase* OR Cinahl* OR systematic overview* OR methodological overview* OR methodologic overview* OR methodological review* OR methodologic review* OR quantitative review* OR quantitative overview* OR quantitative synthes* OR pooled analy* OR Cochrane OR Medline OR Pubmed OR Medlars OR handsearch* OR hand search* OR meta-regression* OR metaregression* OR data synthes* OR data extraction OR data abstraction* OR mantel haenszel OR peto OR der-simonian OR dersimonian OR fixed effect* OR "Cochrane Database Syst Rev" OR "health technology assessment winchester, england" OR "Evid Rep Technol Assess (Full Rep)" OR "Evid Rep Technol Assess (Summ)" OR "Int J Technol Assess Health Care" OR "GMS Health Technol Assess" OR "Health Technol Assess (Rockv)" OR "Health Technol Assess Rep".  #7 #1 OR #2 OR #3 OR #4  #8 #7 AND #5 AND #6 |
| **WOS 17354** |
| #1 TS= (cigarette smoking OR tobacco OR nicotine OR smok* OR cigarette OR tobacco OR nicotine)  #2 TS= (cessation OR cease OR quit* OR abstinence OR abstinent OR stop* OR reject OR reduce OR resist OR resistance OR give up OR withdraw)  #3 TS= (systematic OR meta-analysis OR meta-analysis as topic OR meta-analysis OR meta analy* OR metanaly* OR metaanaly* OR met analy* OR integrative research OR integrative review* OR integrative overview* OR research integration* OR research overview* OR collaborative review* OR collaborative overview* OR systematic review* OR technology assessment* OR technology overview* OR "Technology Assessment, Biomedical" OR HTA OR HTAs OR comparative efficacy OR comparative effectiveness OR outcomes research OR indirect comparison* OR ((indirect treatment OR mixed-treatment) AND comparison*) OR Embase* OR Cinahl* OR systematic overview* OR methodological overview* OR methodologic overview* OR methodological review* OR methodologic review* OR quantitative review* OR quantitative overview* OR quantitative synthes* OR pooled analy* OR Cochrane OR Medline OR Pubmed OR Medlars OR handsearch* OR hand search* OR meta-regression* OR metaregression* OR data synthes* OR data extraction OR data abstraction* OR mantel haenszel OR peto OR der-simonian OR dersimonian OR fixed effect* OR "Cochrane Database Syst Rev" OR "health technology assessment winchester, england" OR "Evid Rep Technol Assess (Full Rep)" OR "Evid Rep Technol Assess (Summ)" OR "Int J Technol Assess Health Care" OR "GMS Health Technol Assess" OR "Health Technol Assess (Rockv)" OR "Health Technol Assess Rep".)  #4 #1 AND #2 AND #3 |
| **Embase 5878** |
| #1 'Cigarette Smoking'/exp OR 'Tobacco'/exp OR 'Nicotine'/exp OR 'smok*'/exp OR 'cigarette'/exp OR 'tobacco'/exp OR 'nicotine'/exp  #2 'cessation'/exp OR 'cease'/exp OR 'quit*'/exp OR 'abstinence'/exp OR 'abstinent'/exp OR 'stop*'/exp OR 'reject'/exp OR 'reduce'/exp OR 'resist'/exp OR 'resistance'/exp OR 'give up'/exp OR 'withdraw'/exp  #3 'meta analysis'/exp OR 'systematic review'/exp OR (meta NEAR/3 analy*):ab,ti OR metaanaly*:ab,ti OR review*:ti OR overview*:ti OR (synthes* NEAR/3 (literature* OR research* OR studies OR data)):ab,ti OR (pooled AND analys*:ab,ti) OR ((data NEAR/2 pool*):ab,ti AND studies:ab,ti) OR medline:ab,ti OR medlars:ab,ti OR embase:ab,ti OR cinahl:ab,ti OR scisearch:ab,ti OR psychinfo:ab,ti OR psycinfo:ab,ti OR psychlit:ab,ti OR psyclit:ab,ti OR cinhal:ab,ti OR cancerlit:ab,ti OR cochrane:ab,ti OR bids:ab,ti OR pubmed:ab,ti OR ovid:ab,ti OR ((hand OR manual OR database* OR computer*) NEAR/2 search*):ab,ti OR (electronic NEAR/2 (database* OR 'data base' OR 'data bases')):ab,ti OR bibliograph*:ab OR 'relevant journals':ab OR ((review* OR overview*) NEAR/10 (systematic* OR methodologic* OR quantitativ* OR research* OR literature* OR studies OR trial* OR effective*)):ab NOT (((retrospective* OR record* OR case* OR patient*) NEAR/2 review*):ab,ti OR ((patient* OR review*) NEAR/2 chart*):ab,ti OR rat:ab,ti OR rats:ab,ti OR mouse:ab,ti OR mice:ab,ti OR hamster:ab,ti OR hamsters:ab,ti OR animal:ab,ti OR animals:ab,ti OR dog:ab,ti OR dogs:ab,ti OR cat:ab,ti OR cats:ab,ti OR bovine:ab,ti OR sheep:ab,ti) NOT ('editorial'/exp OR 'erratum'/de OR 'letter'/exp) NOT ('animal'/exp OR 'nonhuman'/exp NOT ('animal'/exp OR 'nonhuman'/exp AND 'human'/exp))  #4 #1 AND #2 AND #3 |
| **CINAHL 920** |
| #1 SU ( cigarette smoking OR tobacco OR nicotine OR smok* OR cigarette OR tobacco OR nicotine )  #2 SU ( cessation OR cease OR quit* OR abstinence OR abstinent OR stop* OR reject OR reduce OR resist OR resistance OR give up OR withdraw )  #3 SU ( systematic OR meta-analysis OR meta-analysis as topic OR meta-analysis OR meta analy* OR metanaly* OR metaanaly* OR met analy* OR integrative research OR integrative review* OR integrative overview* OR research integration* OR research overview* OR collaborative review* OR collaborative overview* OR systematic review* OR technology assessment* OR technology overview* OR "Technology Assessment, Biomedical" OR HTA OR HTAs OR comparative efficacy OR comparative effectiveness OR outcomes research OR indirect comparison* OR ((indirect treatment OR mixed-treatment) AND comparison*) OR Embase* OR Cinahl* OR systematic overview* OR methodological overview* OR methodologic overview* OR methodological review* OR methodologic review* OR quantitative review* OR quantitative overview* OR quantitative synthes* OR pooled analy* OR Cochrane OR Medline OR Pubmed OR Medlars OR handsearch* OR hand search* OR meta-regression* OR metaregression* OR data synthes* OR data extraction OR data abstraction* OR mantel haenszel OR peto OR der-simonian OR dersimonian OR fixed effect* OR "Cochrane Database Syst Rev" OR "health technology assessment winchester, england" OR "Evid Rep Technol Assess (Full Rep)" OR "Evid Rep Technol Assess (Summ)" OR "Int J Technol Assess Health Care" OR "GMS Health Technol Assess" OR "Health Technol Assess (Rockv)" OR "Health Technol Assess Rep" )  #4 #1AND #2 AND #3 |
| **PsycINFO 194** |
| #1 SU ( cigarette smoking OR tobacco OR nicotine OR smok* OR cigarette OR tobacco OR nicotine )  #2 SU ( cessation OR cease OR quit* OR abstinence OR abstinent OR stop* OR reject OR reduce OR resist OR resistance OR give up OR withdraw )  #3 SU ( systematic OR meta-analysis OR meta-analysis as topic OR meta-analysis OR meta analy* OR metanaly* OR metaanaly* OR met analy* OR integrative research OR integrative review* OR integrative overview* OR research integration* OR research overview* OR collaborative review* OR collaborative overview* OR systematic review* OR technology assessment* OR technology overview* OR "Technology Assessment, Biomedical" OR HTA OR HTAs OR comparative efficacy OR comparative effectiveness OR outcomes research OR indirect comparison* OR ((indirect treatment OR mixed-treatment) AND comparison*) OR Embase* OR Cinahl* OR systematic overview* OR methodological overview* OR methodologic overview* OR methodological review* OR methodologic review* OR quantitative review* OR quantitative overview* OR quantitative synthes* OR pooled analy* OR Cochrane OR Medline OR Pubmed OR Medlars OR handsearch* OR hand search* OR meta-regression* OR metaregression* OR data synthes* OR data extraction OR data abstraction* OR mantel haenszel OR peto OR der-simonian OR dersimonian OR fixed effect* OR "Cochrane Database Syst Rev" OR "health technology assessment winchester, england" OR "Evid Rep Technol Assess (Full Rep)" OR "Evid Rep Technol Assess (Summ)" OR "Int J Technol Assess Health Care" OR "GMS Health Technol Assess" OR "Health Technol Assess (Rockv)" OR "Health Technol Assess Rep" )  #4 #1AND #2 AND #3 |
| **CNKI 74** |
| 吸烟 + 尼古丁 + 烟雾 + 香烟 + 烟草  停止 + 戒烟 + 禁欲 + 拒绝 + 减少 + 抵制 + 抵抗 + 放弃 + 退出  ('Meta 分析' + '系统评价' + '荟萃分析' + '系统综述' + '整合分析' + '数据合成' + '元分析') |
| **CBM 59** |
| #1 "Meta 分析" [常用字段: 智能] OR "系统评价" [常用字段: 智能] OR "荟萃分析" [常用字段: 智能] OR "系统综述" [常用字段: 智能] OR "整合分析" [常用字段: 智能] OR "数据合成" [常用字段: 智能] OR "元分析" [常用字段: 智能]  #2 "吸烟" [常用字段: 智能] OR "尼古丁" [常用字段: 智能] OR "烟雾" [常用字段: 智能] OR "香烟" OR "烟草" [常用字段: 智能]  #3 "停止" [常用字段: 智能] OR "戒烟" [常用字段: 智能] OR "禁欲" [常用字段: 智能] OR "拒绝" OR "减少" [常用字段: 智能] "抵制" [常用字段: 智能] "抵抗" [常用字段: 智能] "放弃" [常用字段: 智能] "退出" [常用字段: 智能]  #1 AND #2 AND #3 |
| **Wangfang 19** |
| 吸烟 or 尼古丁 or 烟雾 or 香烟 or 烟草  停止 or 戒烟 or 禁欲 or 拒绝 or 减少 or 抵制 or 抵抗 or 放弃 or 退出  Meta 分析 or 系统评价 or 荟萃分析 or 系统综述 or 整合分析 or 数据合成 or 元分析 |
| **VIP 9** |
| 吸烟 + 尼古丁 + 烟雾 + 香烟 + 烟草  停止 + 戒烟 + 禁欲 + 拒绝 + 减少 + 抵制 + 抵抗 + 放弃 + 退出  Meta 分析 + 系统评价 + 荟萃分析 + 系统综述 + 整合分析 + 数据合成 + 元分析 |

**Table S2): PRISMA quality appraisal scores**

| **Item No** | 1 | 2 | 3 | 4 | 5 | 6 | 7 | 8 | 9 | 10 | 11 | 12 | 13 | 14 | 15 | 16 | 17 | 18 | 19 | 20 | 21 | 22 | 23 | 24 | 25 | 26 | 27 | Score |
| --- | --- | --- | --- | --- | --- | --- | --- | --- | --- | --- | --- | --- | --- | --- | --- | --- | --- | --- | --- | --- | --- | --- | --- | --- | --- | --- | --- | --- |
| Tzelepis F | N | Y | Y | Y | N | Y | Y | Y | Y | PY | Y | Y | Y | Y | Y | N | Y | Y | Y | Y | Y | Y | N | Y | Y | Y | Y | 22.5 |
| Ravi K | Y | Y | Y | Y | Y | Y | Y | N | Y | PY | Y | Y | N | N | Y | N | N | Y | Y | N | N | Y | N | N | N | Y | Y | 16.5 |
| Byaruhanga J | Y | Y | Y | Y | Y | Y | Y | Y | Y | PY | Y | Y | N | N | N | N | Y | Y | Y | N | N | N | N | Y | Y | Y | Y | 18.5 |
| Oikonomou M | Y | N | Y | Y | N | Y | Y | Y | Y | PY | Y | Y | Y | Y | Y | Y | Y | Y | Y | Y | Y | Y | Y | Y | Y | Y | Y | 24.5 |
| Ybarra ML | Y | N | Y | Y | N | N | N | N | N | N | N | N | Y | N | N | N | N | Y | N | Y | Y | N | N | N | Y | Y | Y | 10 |
| Khoudigian S | Y | Y | Y | Y | N | Y | Y | N | Y | Y | Y | Y | Y | Y | N | Y | Y | Y | Y | Y | Y | N | Y | Y | Y | Y | Y | 23 |
| Hoedjes M | Y | N | Y | Y | N | Y | Y | N | N | N | Y | N | N | N | N | N | Y | Y | N | N | N | N | N | PY | Y | Y | Y | 11.5 |
| Cahill K | N | Y | Y | Y | N | Y | Y | Y | Y | Y | Y | Y | N | N | N | N | Y | Y | Y | N | N | Y | N | PY | Y | Y | Y | 17.5 |
| Rahman MA | Y | Y | Y | Y | N | Y | Y | N | Y | N | N | Y | Y | Y | N | N | Y | Y | Y | Y | Y | Y | N | Y | Y | Y | Y | 20 |
| Gualano MR | Y | Y | Y | Y | N | N | Y | N | Y | N | N | N | N | N | N | N | Y | Y | N | N | N | N | N | PY | Y | Y | N | 10.5 |
| Lancaster T | N | Y | Y | Y | N | Y | Y | N | Y | Y | Y | Y | Y | Y | N | N | Y | Y | Y | Y | Y | N | N | Y | Y | Y | Y | 20 |
| Sinclair HK | N | Y | Y | Y | N | Y | Y | Y | Y | Y | Y | Y | Y | Y | Y | Y | Y | Y | Y | Y | Y | Y | Y | Y | Y | Y | Y | 25 |
| Naslund JA | Y | Y | Y | Y | N | Y | Y | Y | Y | PY | Y | Y | N | N | N | N | Y | Y | Y | N | N | N | N | PY | Y | Y | Y | 17 |
| Haskins BL | Y | N | Y | Y | N | N | Y | N | Y | N | N | N | N | N | N | N | N | N | N | N | N | N | N | N | Y | Y | Y | 8 |
| Rabe GL | Y | Y | Y | Y | N | Y | Y | Y | Y | PY | Y | Y | Y | Y | Y | N | Y | Y | Y | Y | Y | Y | N | Y | Y | Y | Y | 23.5 |
| Gainsbury S | Y | N | Y | Y | N | Y | Y | N | N | N | Y | Y | N | N | N | N | N | Y | Y | N | N | N | N | N | Y | Y | N | 11 |
| McCambridge J | Y | N | Y | N | N | N | Y | N | Y | Y | N | N | Y | N | N | N | N | Y | N | N | Y | N | N | N | Y | Y | Y | 11 |
| Levitt C | Y | Y | Y | N | N | Y | Y | N | Y | Y | Y | Y | N | N | N | N | N | Y | Y | N | N | N | N | N | N | Y | N | 12 |
| Corepal R | Y | N | Y | Y | Y | Y | Y | N | Y | Y | Y | N | Y | Y | N | N | Y | Y | Y | Y | Y | Y | N | Y | Y | Y | Y | 21 |
| Hefler M | N | Y | Y | Y | N | Y | Y | Y | Y | Y | Y | Y | Y | Y | Y | Y | Y | Y | Y | Y | Y | Y | Y | Y | Y | Y | Y | 25 |
| Klemp I | Y | Y | Y | Y | N | Y | Y | N | Y | Y | Y | Y | Y | Y | N | N | Y | Y | N | Y | Y | N | N | PY | Y | Y | N | 18.5 |
| Keith A | Y | Y | Y | Y | N | Y | Y | N | Y | PY | Y | Y | Y | Y | N | N | Y | Y | Y | Y | Y | Y | N | N | Y | Y | N | 19.5 |
| Berlin NL | Y | Y | Y | Y | N | Y | Y | N | Y | PY | Y | N | Y | Y | N | N | Y | Y | Y | Y | Y | N | N | Y | Y | Y | Y | 19.5 |
| Ford P | Y | N | Y | Y | N | N | Y | Y | Y | N | Y | Y | N | N | Y | N | N | N | Y | N | N | N | N | N | N | Y | Y | 12 |
| Hamilton FL | Y | Y | Y | Y | N | Y | Y | N | N | N | Y | Y | N | N | N | N | Y | Y | N | Y | N | N | N | PY | Y | Y | Y | 14.5 |
| Filion KB | Y | N | Y | Y | N | Y | Y | Y | Y | N | Y | Y | N | N | Y | N | N | Y | Y | N | N | Y | N | N | Y | Y | Y | 16 |
| Ussher MH | Y | Y | Y | Y | N | Y | Y | N | Y | PY | Y | N | Y | Y | N | N | Y | Y | N | Y | Y | N | N | Y | Y | Y | Y | 18.5 |
| Wang RJ | Y | Y | Y | Y | N | Y | Y | N | N | N | N | N | N | N | N | N | N | Y | N | N | N | N | N | N | Y | Y | N | 9 |
| Mohamed R | Y | Y | Y | Y | Y | Y | Y | Y | Y | PY | Y | Y | Y | Y | Y | N | Y | Y | Y | Y | Y | Y | N | Y | Y | Y | Y | 24.5 |
| Hussain T | Y | Y | Y | Y | N | Y | Y | N | N | N | N | N | N | Y | N | N | Y | Y | N | Y | Y | N | N | N | N | Y | Y | 13 |
| Cobos-Campos R | PY | Y | Y | Y | Y | Y | Y | Y | Y | PY | Y | Y | N | N | N | N | Y | Y | Y | N | N | N | N | Y | Y | Y | Y | 18 |
| Cobos-Campos R | Y | Y | Y | Y | Y | Y | Y | Y | Y | PY | Y | Y | Y | Y | Y | Y | Y | Y | Y | Y | Y | Y | Y | Y | Y | Y | Y | 26.5 |
| Gentry S | PY | Y | Y | Y | Y | Y | Y | Y | Y | PY | Y | PY | N | N | N | N | Y | Y | N | N | N | N | N | Y | N | Y | PY | 15 |
| Kedzior KK | PY | Y | Y | Y | Y | Y | Y | Y | Y | N | Y | PY | N | N | N | N | Y | Y | N | N | N | N | N | Y | Y | Y | Y | 16 |
| Stead LF | Y | Y | Y | Y | Y | PY | N | Y | N | N | Y | N | Y | Y | N | Y | N | N | N | Y | Y | N | Y | Y | N | Y | Y | 16.5 |
| Gulliver A | Y | Y | Y | Y | Y | Y | Y | Y | PY | Y | PY | Y | Y | PY | N | N | Y | Y | PY | Y | PY | N | N | Y | PY | Y | Y | 20 |
| Trivedi D^37^ | N | Y | Y | Y | Y | Y | Y | Y | Y | Y | Y | Y | Y | Y | Y | Y | Y | Y | Y | Y | Y | Y | Y | Y | Y | Y | Y | 26 |
| van Velthoven MH | PY | Y | Y | Y | Y | Y | Y | Y | Y | Y | Y | PY | N | N | N | N | Y | Y | PY | N | N | N | N | N | PY | Y | Y | 16 |
| Hemsing N | PY | Y | Y | PY | Y | Y | Y | Y | Y | Y | Y | N | N | N | N | N | Y | Y | N | N | N | N | N | PY | PY | Y | Y | 15 |
| Park EW | Y | Y | Y | Y | Y | Y | N | N | PY | Y | Y | PY | Y | Y | N | Y | N | PY | PY | Y | Y | PY | Y | Y | PY | Y | Y | 20 |
| Ashenden R | Y | PY | Y | Y | N | Y | PY | N | PY | Y | PY | Y | Y | Y | PY | N | N | Y | PY | Y | Y | PY | N | N | PY | Y | N | 16 |
| Uthman OA | Y | PY | Y | Y | Y | Y | PY | Y | PY | Y | Y | PY | Y | Y | N | Y | Y | Y | Y | Y | Y | Y | Y | Y | PY | Y | Y | 23.5 |
| Maglione MA | Y | PY | Y | Y | Y | Y | Y | Y | Y | Y | Y | PY | Y | Y | PY | Y | Y | Y | PY | Y | Y | PY | Y | Y | PY | Y | Y | 24 |
| Giles EL | Y | Y | Y | Y | Y | PY | Y | Y | Y | Y | Y | PY | Y | Y | Y | Y | Y | Y | PY | Y | Y | PY | Y | Y | Y | Y | Y | 25 |
| Cheng HM | Y | PY | Y | Y | Y | PY | PY | N | PY | Y | Y | PY | Y | Y | PY | Y | Y | Y | PY | Y | Y | PY | Y | Y | PY | Y | N | 20.5 |
| Spring B | Y | PY | Y | Y | Y | PY | Y | Y | Y | PY | Y | Y | Y | Y | Y | Y | Y | Y | PY | Y | Y | PY | Y | Y | Y | Y | Y | 24.5 |
| May S | PY | PY | Y | Y | Y | PY | Y | PY | N | N | Y | N | N | N | N | N | PY | Y | N | N | N | N | N | Y | PY | Y | N | 11 |
| Smith P | PY | PY | Y | Y | Y | PY | Y | Y | PY | PY | Y | Y | N | N | N | N | PY | Y | PY | N | N | N | N | Y | PY | Y | Y | 15 |
| Livingstone-Banks J | Y | Y | Y | Y | Y | PY | Y | Y | Y | Y | Y | PY | Y | Y | Y | Y | Y | PY | Y | Y | Y | PY | Y | Y | Y | Y | Y | 25 |
| Hopkins DP | PY | PY | Y | Y | Y | Y | N | N | N | Y | Y | N | N | N | N | N | PY | Y | N | N | N | N | N | Y | PY | Y | N | 11 |
| Shahab L | PY | PY | Y | Y | Y | Y | N | Y | PY | Y | Y | N | Y | N | N | N | N | PY | N | Y | N | N | N | Y | PY | Y | Y | 14.5 |
| Krishnan N | PY | Y | Y | Y | Y | Y | PY | PY | Y | Y | Y | Y | N | N | N | N | Y | Y | Y | N | N | PY | N | Y | PY | Y | Y | 17.5 |
| Tseng PT | Y | PY | Y | Y | Y | Y | PY | N | PY | Y | Y | PY | Y | N | PY | N | Y | Y | PY | Y | N | PY | N | Y | Y | Y | Y | 18.5 |
| Grabovac I | Y | Y | Y | Y | Y | Y | PY | Y | Y | Y | Y | N | Y | N | N | N | Y | Y | N | Y | Y | N | N | Y | PY | Y | Y | 19 |
| Griffiths SE | Y | PY | Y | Y | Y | PY | PY | Y | Y | Y | Y | Y | Y | Y | PY | Y | Y | Y | Y | Y | Y | PY | Y | Y | Y | Y | Y | 24.5 |
| Whittaker R | N | Y | Y | Y | Y | Y | Y | Y | Y | Y | Y | Y | Y | Y | Y | Y | Y | Y | Y | Y | Y | Y | Y | Y | Y | Y | N | 25 |
| Park E | PY | PY | Y | Y | Y | PY | PY | Y | PY | Y | Y | N | N | N | N | N | Y | Y | N | N | N | N | N | Y | PY | Y | N | 13 |
| Aziz O | PY | PY | Y | Y | Y | Y | N | N | N | N | PY | N | N | N | N | N | N | Y | N | N | N | N | N | Y | PY | Y | N | 9 |
| Naughton F | Y | PY | Y | Y | Y | Y | PY | Y | PY | Y | PY | Y | Y | Y | Y | Y | Y | Y | Y | Y | Y | Y | Y | Y | PY | Y | PY | 24 |
| Lightfoot K | PY | PY | Y | Y | Y | PY | Y | Y | Y | Y | Y | PY | N | N | N | N | Y | Y | N | N | N | N | N | Y | Y | Y | Y | 16 |
| Secades-Villa R | Y | Y | Y | Y | Y | Y | Y | Y | Y | Y | PY | PY | Y | Y | Y | N | Y | Y | N | Y | Y | N | Y | Y | Y | Y | N | 22 |
| Luo T | PY | Y | Y | Y | N | Y | Y | N | PY | PY | PY | N | N | N | N | N | Y | Y | N | N | N | N | N | Y | Y | Y | Y | 13 |
| Boland VC | Y | Y | Y | Y | N | Y | Y | Y | Y | Y | Y | Y | Y | PY | N | N | Y | Y | Y | Y | Y | Y | N | Y | Y | Y | Y | 22.5 |
| Spohr SA | PY | Y | Y | Y | N | Y | Y | Y | Y | Y | Y | N | Y | Y | Y | N | N | Y | N | Y | Y | N | N | Y | Y | Y | Y | 19.5 |
| White A | PY | Y | Y | Y | N | Y | Y | PY | PY | Y | PY | Y | Y | Y | N | N | Y | PY | Y | Y | Y | N | N | Y | Y | Y | N | 18.5 |
| Lichtenstein E | PY | PY | Y | Y | N | PY | Y | N | N | N | PY | N | Y | Y | N | N | PY | N | N | Y | Y | N | N | Y | N | Y | N | 11.5 |
| Barnes J | N | Y | Y | Y | N | Y | Y | Y | Y | Y | Y | Y | Y | Y | Y | Y | Y | Y | Y | Y | Y | Y | N | Y | Y | Y | Y | 24 |
| Liu X | Y | Y | Y | Y | N | Y | Y | Y | N | Y | PY | PY | Y | Y | N | N | Y | PY | PY | Y | Y | Y | Y | Y | Y | Y | N | 20 |
| Free C | PY | Y | Y | Y | Y | Y | Y | Y | N | Y | Y | Y | Y | Y | Y | N | Y | PY | Y | Y | Y | Y | N | Y | Y | Y | Y | 23 |
| Huttunen-Lenz M | PY | Y | Y | Y | N | Y | Y | Y | Y | Y | Y | PY | Y | Y | Y | Y | Y | PY | PY | Y | Y | Y | Y | Y | Y | N | N | 22 |
| Bar-Zeev Y | Y | Y | Y | Y | Y | Y | Y | Y | Y | Y | Y | PY | Y | Y | Y | Y | Y | Y | Y | Y | Y | Y | Y | Y | Y | Y | Y | 26.5 |
| Hauer L | PY | Y | Y | Y | N | Y | Y | Y | Y | Y | Y | N | N | N | N | N | Y | PY | N | N | N | N | N | Y | Y | Y | N | 14 |
| de Kleijn MJ | PY | Y | Y | Y | Y | Y | Y | Y | Y | Y | Y | PY | Y | Y | Y | Y | Y | PY | Y | Y | Y | Y | Y | Y | Y | Y | Y | 25.5 |
| White AR | PY | Y | Y | Y | N | Y | Y | PY | PY | N | PY | PY | Y | Y | N | N | PY | PY | PY | Y | Y | N | N | Y | Y | Y | N | 16 |
| van den Brand FA | N | Y | Y | Y | N | Y | Y | Y | Y | Y | Y | Y | Y | Y | N | N | Y | Y | Y | Y | Y | N | Y | Y | PY | Y | Y | 21.5 |
| Kazemzadeh Z | PY | Y | Y | Y | N | Y | Y | Y | PY | Y | Y | N | N | N | N | N | Y | PY | N | N | N | N | N | Y | N | PY | N | 12 |
| Bartlett YK | PY | Y | Y | Y | N | Y | Y | Y | N | N | N | PY | Y | Y | Y | N | Y | PY | PY | Y | Y | Y | N | Y | Y | Y | N | 18 |
| Stead LF | N | Y | Y | Y | N | Y | Y | Y | PY | Y | Y | PY | Y | Y | N | N | Y | PY | Y | Y | Y | N | Y | Y | N | Y | Y | 19.5 |
| Carson KV | N | Y | Y | Y | N | Y | Y | Y | Y | Y | Y | Y | Y | Y | Y | Y | Y | Y | Y | Y | Y | Y | Y | Y | N | Y | N | 23 |
| Bull ER | Y | Y | Y | Y | N | Y | Y | Y | Y | Y | Y | Y | Y | Y | Y | Y | Y | Y | Y | Y | Y | Y | Y | Y | Y | Y | Y | 26 |
| Chu KH | PY | Y | Y | Y | Y | Y | Y | Y | Y | Y | Y | Y | N | N | Y | N | Y | Y | Y | Y | N | Y | N | Y | Y | Y | Y | 21.5 |
| Nethan ST | N | Y | Y | Y | N | Y | Y | Y | Y | Y | Y | N | Y | Y | Y | N | Y | Y | N | Y | Y | Y | N | Y | Y | Y | Y | 21 |
| Klinsophon T | PY | Y | Y | Y | N | Y | Y | Y | Y | Y | Y | Y | Y | Y | Y | N | Y | Y | Y | Y | Y | Y | N | Y | Y | Y | Y | 23.5 |
| Haasova M | Y | Y | Y | Y | N | Y | Y | Y | N | N | Y | N | Y | Y | N | N | Y | PY | N | Y | Y | N | N | Y | N | Y | Y | 16.5 |
| Cahill K | N | Y | Y | Y | N | Y | Y | Y | Y | Y | Y | Y | Y | Y | N | N | Y | PY | Y | Y | Y | N | N | Y | N | Y | N | 18.5 |
| Barth J | Y | Y | Y | Y | N | Y | Y | Y | Y | Y | Y | N | Y | Y | N | N | Y | Y | N | Y | Y | N | N | Y | N | Y | N | 18 |
| Hughes JR | PY | Y | Y | PY | N | Y | Y | Y | Y | Y | Y | N | N | N | N | N | Y | Y | N | Y | N | N | N | Y | N | Y | Y | 15 |
| Smedslund G | PY | Y | Y | Y | N | Y | Y | PY | Y | Y | Y | N | Y | Y | N | N | Y | Y | N | Y | Y | Y | N | Y | Y | Y | N | 19 |
| Fanshawe TR | N | Y | Y | Y | N | Y | Y | Y | Y | Y | Y | Y | Y | Y | Y | Y | Y | Y | Y | Y | Y | Y | Y | Y | Y | Y | Y | 25 |
| Scott-Sheldon LA | Y | PY | Y | Y | N | Y | Y | Y | Y | PY | Y | Y | Y | Y | Y | Y | Y | Y | Y | Y | Y | Y | N | Y | Y | Y | N | 23 |
| Bauld L | PY | PY | Y | Y | N | Y | Y | Y | Y | Y | PY | Y | Y | Y | N | N | Y | Y | N | Y | Y | N | N | Y | Y | N | Y | 18.5 |
| Hutton HE | PY | PY | Y | Y | N | Y | Y | Y | Y | Y | PY | Y | Y | Y | N | N | Y | Y | N | Y | Y | N | N | Y | Y | N | Y | 18.5 |
| Wilson SM | PY | PY | Y | Y | N | Y | Y | Y | Y | Y | Y | Y | Y | Y | Y | Y | Y | Y | Y | Y | Y | N | N | Y | N | Y | N | 21 |
| Jiang S | PY | PY | Y | Y | N | Y | Y | Y | Y | Y | Y | Y | Y | Y | N | N | Y | Y | Y | Y | Y | N | N | Y | Y | Y | Y | 21 |
| Prestwich A | Y | Y | Y | Y | Y | Y | Y | Y | Y | Y | Y | PY | Y | Y | Y | Y | Y | Y | N | Y | Y | Y | Y | Y | Y | Y | Y | 25.5 |
| Williams MT | PY | PY | Y | Y | Y | Y | Y | Y | Y | Y | Y | PY | Y | Y | Y | N | Y | Y | N | Y | Y | N | N | Y | N | Y | N | 19.5 |
| Myung SK | PY | PY | Y | Y | N | Y | Y | Y | Y | Y | Y | Y | Y | Y | Y | Y | Y | Y | N | Y | Y | Y | Y | Y | Y | N | Y | 23 |
| Pan W | PY | PY | Y | Y | N | Y | Y | Y | Y | PY | PY | PY | Y | Y | Y | N | Y | Y | Y | Y | Y | Y | Y | Y | Y | N | N | 20.5 |
| Shoesmith E | PY | PY | Y | Y | Y | Y | Y | Y | Y | Y | PY | PY | Y | Y | N | N | Y | PY | Y | Y | Y | N | N | Y | Y | N | N | 18.5 |
| Ussher MH | N | Y | Y | Y | N | Y | Y | Y | PY | Y | Y | Y | Y | Y | Y | N | Y | Y | Y | Y | Y | Y | N | Y | Y | Y | N | 21.5 |
| Wang JH | Y | PY | Y | Y | N | Y | Y | Y | Y | Y | Y | Y | Y | Y | Y | N | Y | Y | Y | Y | Y | N | Y | Y | Y | Y | N | 22.5 |
| Wilson A | PY | PY | Y | Y | N | Y | Y | Y | Y | Y | Y | Y | Y | Y | N | N | Y | PY | Y | Y | Y | N | N | Y | Y | Y | N | 19.5 |
| Tzelepis F | PY | PY | Y | Y | N | Y | Y | Y | PY | Y | Y | Y | Y | Y | Y | Y | Y | Y | Y | Y | Y | N | N | Y | Y | N | N | 20.5 |
| Fichtenberg CM | PY | Y | Y | Y | N | Y | Y | Y | Y | PY | PY | PY | Y | Y | Y | N | Y | Y | N | Y | Y | Y | Y | Y | Y | N | N | 20 |
| Di YM | PY | PY | Y | Y | N | Y | Y | Y | Y | Y | PY | PY | Y | Y | Y | Y | Y | Y | N | Y | Y | N | N | Y | N | Y | Y | 20 |
| Hajek P | N | Y | Y | Y | N | Y | Y | Y | PY | Y | PY | PY | Y | Y | N | N | Y | Y | Y | Y | Y | Y | Y | Y | Y | Y | N | 20.5 |
| Lancaster T | N | Y | Y | Y | N | Y | Y | Y | PY | Y | Y | PY | Y | Y | Y | N | Y | Y | Y | Y | Y | Y | Y | Y | N | Y | N | 21 |
| Hyndman K | Y | PY | Y | Y | Y | Y | Y | Y | Y | Y | Y | Y | Y | Y | Y | Y | Y | PY | N | Y | Y | N | N | Y | Y | Y | Y | 23 |
| Lindson N | PY | Y | Y | Y | N | Y | Y | Y | PY | Y | Y | Y | Y | Y | Y | N | Y | Y | Y | Y | Y | Y | Y | Y | Y | Y | N | 23 |
| Hettema JE | Y | PY | Y | Y | N | Y | Y | Y | PY | PY | PY | Y | Y | Y | Y | Y | Y | Y | Y | Y | Y | Y | N | Y | N | N | N | 20 |
| Heckman CJ | Y | Y | Y | Y | N | Y | Y | Y | PY | Y | Y | PY | Y | Y | Y | Y | Y | Y | N | Y | Y | Y | N | Y | Y | N | Y | 22 |
| Choi Y | Y | PY | Y | Y | N | Y | Y | Y | PY | Y | Y | Y | Y | Y | Y | Y | Y | Y | N | Y | Y | Y | Y | Y | Y | Y | N | 23 |
| Bryant J | Y | PY | Y | Y | N | Y | Y | Y | Y | Y | Y | Y | Y | Y | Y | N | Y | Y | Y | Y | Y | N | N | Y | Y | Y | N | 21.5 |
| Bafunno D | PY | PY | Y | Y | N | Y | Y | Y | Y | Y | PY | Y | Y | Y | N | N | Y | Y | N | Y | Y | N | N | Y | Y | Y | N | 18.5 |
| Rice VH | PY | PY | Y | Y | N | Y | Y | Y | PY | Y | Y | Y | Y | Y | Y | N | Y | Y | Y | Y | Y | N | N | Y | N | Y | N | 19.5 |
| Rice VH | N | Y | Y | Y | N | Y | Y | Y | PY | Y | Y | Y | Y | Y | Y | Y | Y | Y | N | Y | Y | Y | N | Y | Y | N | N | 20.5 |
| Wray JM | PY | PY | Y | Y | N | Y | Y | Y | Y | Y | PY | PY | Y | Y | Y | Y | Y | Y | Y | Y | Y | N | N | Y | Y | Y | Y | 22 |
| Kelley K | PY | Y | Y | Y | N | Y | Y | Y | PY | PY | PY | PY | Y | Y | N | N | Y | Y | N | Y | Y | Y | Y | Y | Y | N | N | 18.5 |
| Papadakis S | Y | PY | Y | Y | N | Y | Y | Y | Y | Y | Y | PY | Y | Y | Y | Y | Y | Y | N | Y | Y | Y | Y | Y | Y | Y | N | 23 |
| Gorin SS | PY | N | Y | Y | N | Y | Y | Y | PY | PY | Y | N | Y | Y | N | Y | Y | Y | N | Y | Y | N | Y | Y | N | Y | Y | 18.5 |
| Kalkhoran S | Y | Y | Y | Y | Y | Y | Y | Y | Y | Y | Y | N | Y | Y | N | Y | Y | Y | N | Y | Y | N | Y | Y | Y | Y | Y | 23 |
| White AR | N | Y | Y | Y | Y | Y | Y | Y | Y | Y | Y | Y | Y | Y | Y | N | Y | Y | Y | Y | Y | Y | N | Y | Y | Y | Y | 24 |
| Graham AL | Y | Y | Y | Y | N | Y | Y | Y | Y | Y | Y | Y | Y | Y | Y | N | Y | Y | Y | Y | Y | Y | N | Y | N | Y | Y | 23 |
| Kock L | Y | PY | Y | Y | N | Y | Y | Y | Y | Y | Y | N | Y | Y | N | Y | Y | Y | N | N | Y | Y | Y | Y | Y | Y | Y | 21.5 |
| McCrabb S | Y | Y | Y | Y | Y | Y | Y | Y | Y | Y | Y | Y | Y | Y | Y | Y | Y | Y | Y | Y | Y | Y | Y | Y | Y | Y | Y | 27 |
| Sussman S | PY | PY | Y | Y | N | Y | Y | Y | PY | PY | PY | N | Y | Y | N | N | Y | N | N | N | Y | N | N | Y | Y | Y | N | 14.5 |
| Hartmann-Boyce J | N | Y | Y | Y | Y | Y | Y | Y | Y | Y | Y | Y | Y | Y | Y | Y | Y | Y | Y | Y | Y | Y | Y | Y | Y | Y | Y | 26 |
| Malas M | PY | PY | Y | Y | Y | Y | Y | Y | Y | Y | Y | Y | Y | Y | Y | N | Y | Y | Y | Y | Y | Y | N | Y | Y | Y | N | 23 |
| Matkin W | N | Y | Y | Y | Y | Y | Y | Y | Y | Y | Y | Y | Y | Y | Y | Y | Y | Y | Y | Y | Y | Y | Y | Y | Y | Y | Y | 26 |
| Taylor GM | N | Y | Y | Y | Y | Y | Y | Y | Y | Y | Y | Y | Y | Y | Y | Y | Y | Y | Y | Y | Y | Y | Y | Y | Y | Y | Y | 26 |
| Adamson A | Y | PY | Y | Y | N | Y | Y | Y | PY | PY | PY | N | Y | Y | N | N | Y | Y | N | Y | Y | N | N | Y | N | Y | Y | 17 |
| Darabseh MZ | Y | Y | Y | Y | Y | Y | Y | Y | Y | Y | Y | Y | Y | Y | Y | N | Y | Y | Y | Y | Y | Y | N | Y | Y | Y | Y | 25 |
| Li J | Y | PY | Y | Y | N | Y | Y | Y | Y | Y | Y | Y | Y | Y | Y | Y | Y | Y | Y | Y | Y | Y | Y | Y | Y | Y | Y | 25.5 |
| Kim MM | Y | Y | Y | Y | Y | Y | Y | Y | Y | Y | Y | Y | Y | Y | Y | Y | Y | Y | Y | Y | Y | Y | Y | Y | Y | Y | Y | 27 |
| Schöttl SE | Y | Y | Y | Y | Y | Y | Y | Y | Y | Y | Y | Y | Y | Y | Y | Y | Y | Y | Y | Y | Y | Y | Y | Y | Y | Y | Y | 27 |
| Petit B | Y | Y | Y | Y | Y | Y | Y | Y | Y | Y | Y | Y | Y | Y | Y | Y | Y | Y | Y | Y | Y | Y | Y | Y | N | Y | Y | 26 |
| Jackson S | Y | Y | Y | Y | Y | Y | Y | Y | Y | Y | Y | Y | Y | Y | Y | Y | Y | Y | Y | Y | Y | Y | Y | Y | Y | Y | Y | 27 |
| Saroj SK | Y | PY | Y | Y | N | Y | Y | Y | PY | Y | Y | Y | Y | Y | N | N | Y | Y | Y | Y | N | N | N | Y | Y | Y | Y | 20 |
| Hanewinkel R | Y | Y | Y | Y | Y | Y | Y | Y | Y | Y | Y | Y | Y | Y | Y | Y | Y | Y | Y | Y | Y | Y | Y | Y | Y | Y | Y | 27 |
| Lee EJJJoad | Y | PY | Y | Y | N | Y | Y | Y | PY | Y | Y | Y | Y | N | N | N | Y | Y | Y | N | N | N | N | Y | N | Y | Y | 17 |
| Do HP | Y | Y | Y | Y | Y | Y | Y | Y | Y | Y | Y | Y | Y | Y | Y | N | Y | Y | Y | Y | Y | N | N | Y | Y | Y | Y | 24 |
| Chen H | Y | PY | Y | Y | N | Y | Y | Y | Y | Y | Y | Y | N | N | N | N | Y | Y | N | N | N | N | N | Y | Y | Y | Y | 16.5 |
| García-Fernández G | Y | PY | Y | Y | N | Y | Y | Y | Y | Y | Y | Y | Y | Y | Y | Y | Y | Y | Y | Y | Y | Y | Y | Y | Y | Y | Y | 25.5 |
| Peng Shi | Y | PY | Y | Y | N | Y | Y | Y | Y | Y | Y | Y | Y | N | Y | Y | Y | Y | Y | Y | N | N | Y | Y | Y | Y | Y | 22.5 |
| Hongjun Kuang | Y | PY | Y | Y | N | Y | Y | Y | Y | Y | Y | Y | Y | Y | Y | Y | Y | Y | Y | Y | Y | Y | Y | Y | Y | Y | Y | 25.5 |

Y: Yes; PY: Part of yes; N: no; Item 1: Identify the report as a systematic review. Item 2: See the PRISMA 2020 for Abstracts checklist. Item 3: Describe the rationale for the review in the context of existing knowledge. Item 4: Provide an explicit statement of the objective(s) or question(s) the review addresses. Item 5: Specify the inclusion and exclusion criteria for the review and how studies were grouped for the syntheses. Item 6: Specify all databases, registers, websites, organisations, reference lists and other sources searched or consulted to identify studies. Specify the date when each source was last searched or consulted. Item 7: Present the full search strategies for all databases, registers and websites, including any filters and limits used. Item 8: Specify the methods used to decide whether a study met the inclusion criteria of the review, including how many reviewers screened each record and each report retrieved, whether they worked independently, and if applicable, details of automation tools used in the process. Item 9: Specify the methods used to collect data from reports, including how many reviewers collected data from each report, whether they worked independently, any processes for obtaining or confirming data from study investigators, and if applicable, details of automation tools used in the process. Item 10a: List and define all outcomes for which data were sought. Specify whether all results that were compatible with each outcome domain in each study were sought (e.g. for all measures, time points, analyses), and if not, the methods used to decide which results to collect. Item 10b: List and define all other variables for which data were sought (e.g. participant and intervention characteristics, funding sources). Describe any assumptions made about any missing or unclear information. Item 11: Specify the methods used to assess risk of bias in the included studies, including details of the tool(s) used, how many reviewers assessed each study and whether they worked independently, and if applicable, details of automation tools used in the process. Item 12: Specify for each outcome the effect measure(s) (e.g. risk ratio, mean difference) used in the synthesis or presentation of results. Item 13: Describe the processes used to decide which studies were eligible for each synthesis (e.g. tabulating the study intervention characteristics and comparing against the planned groups for each synthesis (item #5)). Item 14: Describe any methods used to assess risk of bias due to missing results in a synthesis (arising from reporting biases). Item 15: Describe any methods used to assess certainty (or confidence) in the body of evidence for an outcome. Item 16: Describe the results of the search and selection process, from the number of records identified in the search to the number of studies included in the review, ideally using a flow diagram. Item 17: Cite each included study and present its characteristics. Item 18: Present assessments of risk of bias for each included study. Item 19: For all outcomes, present, for each study: (a) summary statistics for each group (where appropriate) and (b) an effect estimate and its precision (e.g. confidence/credible interval), ideally using structured tables or plots. Item 20: For each synthesis, briefly summarise the characteristics and risk of bias among contributing studies. Item 21: Present assessments of risk of bias due to missing results (arising from reporting biases) for each synthesis assessed. Item 22: Present assessments of certainty (or confidence) in the body of evidence for each outcome assessed. Item 23: Provide a general interpretation of the results in the context of other evidence. Item 24: Provide registration information for the review, including register name and registration number, or state that the review was not registered. Item 25: Describe sources of financial or non-financial support for the review, and the role of the funders or sponsors in the review. Item 26: Declare any competing interests of review authors. Item 27: Report which of the following are publicly available and where they can be found: template data collection forms; data extracted from included studies; data used for all analyses; analytic code; any other materials used in the review.

**Table S3): AMSTAR 2 quality appraisal scores**

| **Item No** | **1** | **2*** | **3** | **4*** | **5** | **6** | **7*** | **8** | **9*** | **10** | **11*** | **12** | **13*** | **14** | **15*** | **16** | **Overall Rating** |
| --- | --- | --- | --- | --- | --- | --- | --- | --- | --- | --- | --- | --- | --- | --- | --- | --- | --- |
| Tzelepis F | Y | N | N | Y | Y | Y | Y | Y | Y | Y | Y | Y | Y | Y | N | Y | Low |
| Ravi K | Y | PY | N | PY | Y | Y | N | PY | Y | N | NA | NA | N | N | NA | Y | Critically Low |
| Byaruhanga J | Y | PY | Y | PY | Y | Y | N | PY | Y | N | NA | NA | N | N | NA | Y | Critically Low |
| Oikonomou MT | Y | N | N | PY | Y | Y | N | PY | Y | N | Y | N | Y | N | Y | Y | Critically Low |
| Ybarra ML | N | N | N | N | N | N | N | PY | N | N | Y | N | N | N | N | Y | Critically Low |
| Khoudigian S | Y | N | N | Y | Y | Y | N | PY | Y | N | Y | N | N | Y | N | Y | Critically Low |
| Hoedjes M | Y | N | N | N | N | N | N | PY | N | N | NA | NA | N | N | NA | Y | Critically Low |
| Cahill K | Y | N | N | Y | Y | Y | Y | Y | Y | N | NA | Y | N | N | NA | Y | Critically Low |
| Rahman MA | Y | N | N | PY | N | N | N | PY | Y | N | Y | N | N | Y | N | Y | Critically Low |
| Gualano MR | N | N | N | PY | N | N | N | PY | N | Y | NA | N | N | N | NA | N | Critically Low |
| Lancaster T | Y | N | N | PY | Y | Y | Y | PY | Y | N | Y | N | Y | Y | N | Y | Critically Low |
| Sinclair HK | Y | N | N | Y | Y | Y | Y | Y | Y | Y | Y | Y | Y | N | Y | Y | Low |
| Naslund JA | Y | N | N | PY | Y | Y | N | Y | Y | N | NA | NA | N | N | NA | Y | Critically Low |
| Haskins BL | N | N | N | PY | Y | N | N | N | N | N | NA | NA | N | N | NA | Y | Critically Low |
| Rabe GL | Y | N | N | PY | Y | Y | N | PY | Y | N | Y | Y | Y | N | N | Y | Critically Low |
| Gainsbury S | N | N | N | PY | N | N | N | PY | PY | N | NA | NA | N | N | NA | N | Critically Low |
| McCambridge J | N | N | N | PY | N | N | N | PY | N | N | Y | N | N | N | N | Y | Critically Low |
| Levitt C | N | N | N | PY | N | N | N | PY | PY | N | NA | NA | N | N | NA | N | Critically Low |
| Corepal R | Y | PY | N | PY | Y | N | N | Y | Y | N | Y | Y | Y | Y | N | Y | Critically Low |
| Hefler M | Y | N | N | Y | Y | Y | Y | Y | Y | N | Y | Y | Y | Y | Y | Y | Low |
| Klemp I | Y | N | N | PY | N | N | N | PY | N | N | Y | N | N | Y | N | N | Critically Low |
| Keith A | Y | N | N | PY | Y | Y | N | PY | Y | N | Y | Y | Y | Y | N | N | Critically Low |
| Berlin NL | Y | N | N | PY | Y | Y | N | PY | Y | N | Y | Y | Y | Y | N | Y | Critically Low |
| Ford P | N | N | N | PY | Y | N | N | N | Y | N | NA | NA | N | N | NA | Y | Critically Low |
| Hamilton FL | N | N | N | PY | N | N | N | PY | Y | N | NA | NA | N | N | NA | Y | Critically Low |
| Filion KB | N | N | N | PY | Y | Y | N | PY | N | N | Y | N | N | N | N | Y | Critically Low |
| Ussher MH | N | N | N | PY | N | N | N | PY | N | N | NA | NA | N | N | NA | N | Critically Low |
| Wang RJ | Y | Y | N | PY | Y | Y | N | PY | Y | N | Y | Y | Y | Y | Y | Y | Low |
| Mohamed R | Y | N | N | PY | N | N | N | PY | N | N | Y | N | N | N | N | Y | Critically Low |
| Hussain T | Y | PY | Y | Y | Y | Y | PY | Y | Y | N | NA | N | Y | N | NA | Y | Moderate |
| Cobos-Campos R | Y | Y | Y | Y | N | N | PY | Y | Y | N | Y | Y | Y | Y | Y | Y | Moderate |
| Cobos-Campos R | N | N |  | PY | Y | N | N | PY | Y | N | NA | NA | N | N | NA | Y | Critically Low |
| Gentry S | Y | Y | Y | Y | Y | Y | PY | Y | Y | N | NA | NA | Y | Y | NA | Y | Critically Low |
| Kedzior KK | Y | PY | Y | PY | Y | Y | PY | Y | Y | N | NA | NA | N | N | NA | Y | Low |
| Stead LF | Y | Y | Y | Y | Y | Y | N | N | N | N | Y | N | N | Y | N | Y | Critically Low |
| Gulliver A | Y | Y | Y | PY | Y | Y | PY | Y | Y | N | NA | N | N | N | N | Y | Critically Low |
| Trivedi D | Y | Y | Y | Y | Y | Y | Y | Y | Y | N | Y | Y | Y | Y | Y | Y | High |
| van Velthoven MH | Y | Y | Y | Y | Y | Y | PY | Y | Y | N | NA | N | N | N | NA | Y | Low |
| Hemsing N | Y | Y | Y | PY | Y | Y | PY | Y | N | N | NA | N | N | N | NA | Y | Critically Low |
| Park EW | Y | Y | Y | Y | Y | Y | N | PY | Y | N | Y | Y | Y | Y | N | Y | Critically Low |
| Ashenden R | Y | N | N | Y | N | Y | PY | Y | PY | N | Y | Y | Y | Y | N | N | Critically Low |
| Uthman OA | Y | Y | Y | Y | Y | Y | PY | Y | Y | N | Y | Y | N | Y | N | Y | Critically Low |
| Maglione MA | Y | Y | Y | Y | Y | Y | Y | Y | Y | N | Y | Y | Y | Y | N | Y | Low |
| Giles EL | Y | Y | Y | Y | Y | Y | Y | PY | Y | N | Y | Y | Y | Y | Y | Y | High |
| Cheng HM | Y | Y | Y | Y | Y | Y | PY | Y | PY | N | Y | Y | Y | Y | N | N | Low |
| Spring B | Y | Y | Y | Y | Y | Y | Y | Y | PY | N | Y | Y | Y | Y | Y | Y | High |
| May S | Y | PY | Y | PY | N | N | N | Y | N | N | NA | NA | Y | N | NA | N | Low |
| Smith P | Y | PY | Y | PY | N | N | N | Y | PY | N | NA | NA | Y | N | NA | Y | Moderate |
| Livingstone-Banks J | Y | Y | Y | PY | Y | Y | Y | Y | Y | N | Y | Y | Y | Y | N | Y | Low |
| Hopkins DP | Y | PY | Y | Y | Y | Y | N | PY | N | N | NA | NA | N | N | NA | N | Critically Low |
| Shahab L | Y | Y | Y | Y | Y | Y | PY | PY | PY | N | Y | Y | Y | Y | N | Y | Low |
| Krishnan N | Y | N | Y | Y | Y | Y | N | Y | PY | N | NA | NA | N | N | NA | Y | Critically Low |
| Tseng PT | Y | Y | Y | PY | Y | Y | PY | Y | Y | N | Y | Y | Y | Y | N | Y | Low |
| Grabovac I | Y | Y | Y | PY | Y | Y | Y | Y | N | N | Y | N | N | Y | N | Y | Critically Low |
| Griffiths SE | Y | Y | Y | Y | Y | Y | PY | PY | Y | N | Y | Y | Y | Y | Y | Y | Critically Low |
| Whittaker R | Y | Y | Y | Y | Y | Y | Y | P | Y | N | Y | Y | Y | Y | N | Y | Low |
| Park E | Y | PY | Y | Y | Y | Y | PY | P | N | N | NA | NA | N | N | NA | N | Critically Low |
| Aziz O | Y | Y | N | PY | N | N | PY | P | N | N | NA | NA | Y | Y | NA | N | Critically Low |
| Naughton F | Y | Y | Y | Y | Y | Y | PY | P | Y | N | Y | Y | Y | Y | N | Y | Low |
| Lightfoot K | Y | PY | Y | PY | Y | Y | PY | P | Y | N | NA | NA | Y | N | NA | Y | Moderate |
| Secades-Villa R | Y | Y | Y | Y | Y | Y | N | P | N | N | Y | N | N | N | Y | N | Critically Low |
| Luo T | Y | N | N | N | N | N | N | PY | N | N | NA | NA | N | N | NA | Y | Critically Low |
| Boland VC | Y | N | N | PY | Y | Y | N | Y | Y | N | Y | N | Y | Y | N | Y | Critically Low |
| Spohr SA | Y | N | N | Y | Y | Y | N | Y | N | N | Y | N | N | N | Y | Y | Critically Low |
| White A | Y | N | Y | PY | N | Y | N | PY | Y | N | Y | N | Y | N | N | N | Critically Low |
| Lichtenstein E | Y | N | N | N | N | N | N | N | N | N | Y | N | N | Y | N | N | Critically Low |
| Barnes J | Y | N | N | PY | Y | Y | Y | Y | Y | Y | Y | Y | Y | N | N | Y | Critically Low |
| Liu X | Y | N | N | PY | N | Y | N | PY | PY | N | NA | NA | N | N | NA | N | Critically Low |
| Free C | Y | Y | N | PY | Y | Y | Y | PY | Y | N | Y | NA | N | N | Y | Y | Low |
| Huttunen-Lenz M | Y | N | N | PY | Y | Y | N | PY | PY | N | Y | N | N | N | Y | N | Critically Low |
| Bar-Zeev Y | Y | Y | N | PY | Y | Y | N | Y | Y | N | Y | Y | N | N | Y | Y | Critically Low |
| Hauer L | Y | N | N | PY | Y | Y | N | PY | N | N | NA | NA | N | N | NA | N | Critically Low |
| de Kleijn MJ | Y | Y | N | PY | Y | Y | N | PY | Y | N | Y | N | N | N | Y | Y | Critically Low |
| White AR | Y | N | N | Y | N | N | N | PY | PY | N | Y | N | N | N | N | N | Critically Low |
| van den Brand FA | Y | N | N | PY | Y | Y | N | PY | Y | N | Y | N | N | N | N | Y | Critically Low |
| Kazemzadeh Z | Y | N | N | PY | N | Y | N | Y | N | N | NA | NA | N | N | NA | N | Critically Low |
| Bartlett YK | Y | N | N | PY | N | N | N | PY | PY | N | Y | N | Y | N | Y | N | Critically Low |
| Stead LF | Y | N | N | PY | N | N | N | PY | Y | N | Y | N | Y | Y | N | Y | Critically Low |
| Carson KV | Y | N | N | Y | Y | Y | Y | Y | Y | N | Y | N | Y | Y | Y | N | Low |
| Bull ER | Y | N | N | PY | Y | Y | N | PY | Y | N | Y | N | Y | Y | Y | Y | Critically Low |
| Chu KH | Y | Y | N | PY | N | N | N | PY | PY | Y | NA | NA | N | N | NA | Y | Critically Low |
| Nethan ST | Y | Y | N | PY | Y | Y | N | PY | N | N | Y | N | Y | Y | Y | Y | Critically Low |
| Klinsophon T | Y | N | N | Y | N | Y | N | Y | Y | N | Y | N | Y | N | Y | Y | Critically Low |
| Haasova M | Y | N | N | Y | N | N | N | PY | N | N | Y | N | N | N | N | Y | Critically Low |
| Cahill K | Y | N | N | Y | Y | Y | N | PY | Y | N | Y | N | N | N | N | N | Critically Low |
| Barth J | Y | N | N | Y | N | Y | Y | PY | N | N | Y | N | N | Y | Y | N | Critically Low |
| Hughes JR | Y | N | N | Y | N | Y | N | PY | N | N | NA | NA | N | N | NA | Y | Critically Low |
| Smedslund G | Y | N | N | PY | Y | Y | N | PY | N | N | Y | N | Y | N | Y | N | Critically Low |
| Fanshawe TR | Y | Y | N | Y | Y | Y | Y | Y | Y | Y | Y | Y | Y | Y | Y | N | High |
| Scott-Sheldon LA | Y | N | N | Y | N | N | N | PY | N | N | Y | N | N | Y | Y | Y | Critically Low |
| Bauld L | Y | PY | N | Y | N | N | N | Y | Y | N | NA | NA | Y | N | NA | Y | Critically Low |
| Hutton HE | Y | N | N | Y | Y | Y | N | PY | Y | N | NA | NA | Y | Y | NA | Y | Critically Low |
| Wilson SM | Y | N | N | Y | Y | Y | N | Y | Y | N | Y | Y | Y | Y | NA | N | Critically Low |
| Jiang S | Y | N | N | Y | Y | Y | N | PY | Y | N | NA | NA | Y | Y | NA | Y | Critically Low |
| Prestwich A | Y | Y | Y | Y | Y | Y | N | Y | N | N | Y | N | N | Y | Y | Y | Critically Low |
| Williams MT | Y | Y | Y | Y | Y | Y | N | Y | Y | N | NA | NA | Y | Y | NA | Y | Critically Low |
| Myung SK | Y | Y | Y | Y | Y | Y | PY | Y | Y | N | Y | Y | Y | Y | Y | Y | Low |
| Pan W | Y | N | N | Y | Y | Y | Y | Y | Y | N | Y | Y | Y | Y | Y | Y | Moderate |
| Shoesmith E | Y | Y | N | Y | Y | Y | N | Y | Y | N | Y | Y | Y | Y | N | Y | Critically Low |
| Ussher MH | Y | Y | Y | Y | Y | Y | Y | Y | Y | Y | Y | Y | Y | Y | Y | Y | High |
| Wang JH | Y | N | N | Y | Y | Y | N | Y | Y | N | Y | Y | Y | Y | Y | Y | Critically Low |
| Wilson A | Y | N | N | Y | Y | Y | N | Y | Y | N | NA | NA | Y | Y | NA | Y | Low |
| Tzelepis F | Y | N | Y | Y | Y | Y | N | Y | Y | N | Y | Y | Y | Y | N | Y | Critically Low |
| Fichtenberg CM | Y | N | N | Y | N | N | Y | Y | N | N | NA | NA | Y | Y | NA | N | Critically Low |
| Di YM | Y | N | N | Y | Y | Y | N | Y | Y | N | Y | Y | Y | Y | N | Y | Critically Low |
| Hajek P | Y | Y | Y | Y | Y | Y | Y | Y | Y | N | Y | Y | Y | Y | N | Y | Moderate |
| Lancaster T | Y | Y | Y | Y | Y | Y | Y | Y | Y | N | Y | Y | Y | Y | Y | Y | High |
| Hyndman K | Y | Y | Y | Y | Y | Y | Y | Y | Y | N | Y | Y | Y | Y | N | Y | Low |
| Lindson N | Y | Y | Y | Y | Y | Y | Y | Y | Y | Y | Y | Y | Y | Y | Y | Y | High |
| Hettema JE | Y | N | N | Y | Y | Y | N | Y | Y | N | Y | Y | Y | Y | Y | N | Critically Low |
| Heckman CJ | Y | N | Y | Y | Y | Y | Y | PY | N | N | Y | N | N | Y | Y | Y | Critically Low |
| Choi Y | Y | N | N | Y | Y | Y | N | Y | Y | N | Y | Y | Y | Y | N | Y | Critically Low |
| Bryant J | Y | N | N | Y | Y | Y | N | Y | Y | N | Y | Y | Y | Y | N | Y | Critically Low |
| Bafunno D | Y | N | N | Y | N | N | N | Y | N | N | NA | NA | N | N | NA | N | Critically Low |
| Rice VH | Y | N | Y | Y | Y | Y | N | Y | Y | N | Y | Y | Y | Y | N | N | Critically Low |
| Rice VH | Y | Y | Y | Y | Y | Y | Y | Y | N | N | Y | N | N | Y | N | Y | Critically Low |
| Wray JM | Y | N | N | N | N | N | N | PY | Y | N | Y | Y | Y | Y | Y | Y | Critically Low |
| Kelley K | Y | N | N | PY | N | N | N | PY | N | N | Y | N | N | N | N | N | Critically Low |
| Papadakis S | Y | N | Y | N | Y | Y | N | Y | Y | N | Y | Y | Y | Y | N | Y | Critically Low |
| Gorin SS | Y | N | Y | Y | N | N | N | Y | N | N | Y | N | N | Y | Y | Y | Critically Low |
| Kalkhoran S | Y | Y | N | Y | Y | Y | PY | Y | N | N | Y | N | N | Y | Y | Y | Critically Low |
| White AR | Y | Y | Y | Y | Y | Y | Y | Y | Y | Y | Y | Y | Y | Y | Y | Y | High |
| Graham AL | Y | N | Y | Y | Y | Y | Y | Y | Y | N | Y | Y | Y | Y | N | Y | Critically Low |
| Kock L | Y | N | N | Y | Y | Y | N | Y | N | N | Y | N | N | Y | N | Y | Critically Low |
| McCrabb S | Y | Y | Y | Y | Y | Y | N | Y | Y | Y | Y | Y | Y | Y | Y | Y | Low |
| Sussman S | Y | N | N | Y | N | N | PY | N | N | N | Y | N | N | N | N | N | Critically Low |
| Hartmann-Boyce J | Y | Y | Y | Y | Y | Y | Y | Y | Y | Y | Y | Y | Y | Y | Y | Y | High |
| Malas M | Y | Y | N | Y | Y | Y | N | PY | Y | N | Y | Y | Y | N | N | N | Critically Low |
| Matkin W | Y | Y | Y | Y | Y | Y | Y | Y | Y | Y | Y | Y | Y | Y | Y | Y | High |
| Taylor GM | Y | Y | Y | Y | Y | Y | Y | Y | Y | Y | Y | Y | Y | Y | Y | Y | High |
| Adamson A | Y | N | N | Y | Y | Y | N | PY | Y | N | Y | Y | N | N | Y | Y | Critically Low |
| Darabseh MZ | Y | PY | N | Y | Y | Y | N | Y | Y | N | Y | Y | N | Y | Y | Y | Low |
| Li J | Y | N | N | Y | Y | Y | N | Y | N | N | Y | N | N | N | Y | Y | Critically Low |
| Kim MM | Y | N | N | Y | Y | Y | N | Y | Y | N | Y | Y | Y | N | Y | Y | Low |
| Schöttl SE | Y | Y | N | Y | Y | Y | N | Y | Y | N | NA | NA | Y | Y | NA | Y | Low |
| Petit B | Y | N | N | Y | Y | Y | N | Y | Y | N | Y | Y | Y | N | Y | Y | Low |
| Jackson S | Y | Y | Y | Y | Y | Y | Y | Y | Y | Y | Y | Y | Y | Y | Y | Y | High |
| Saroj SK | Y | Y | N | Y | Y | Y | N | Y | Y | N | NA | NA | Y | N | NA | Y | Low |
| Hanewinkel R | Y | Y | N | Y | Y | Y | PY | Y | Y | Y | Y | Y | Y | Y | Y | Y | Moderate |
| Lee EJJJoad | Y | Y | N | Y | Y | Y | N | Y | Y | N | Y | Y | Y | Y | Y | Y | Moderate |
| Do HP | Y | Y | N | Y | Y | Y | PY | Y | Y | N | Y | Y | Y | Y | Y | Y | Moderate |
| Chen H | Y | N | N | Y | Y | Y | N | Y | Y | N | NA | NA | N | N | NA | Y | Critically Low |
| García-Fernández G | Y | Y | N | Y | Y | Y | Y | Y | Y | N | NA | NA | Y | Y | NA | Y | Moderate |
| Peng Shi | Y | N | N | PY | Y | Y | N | PY | Y | N | Y | N | N | N | Y | Y | Critically Low |
| Hongjun Kuang | Y | N | N | PY | Y | Y | N | Y | Y | N | Y | N | N | N | Y | Y | Critically Low |

*Critical items identified in the AMSTAR2 scale; Y: Yes; PY: Part of yes; N: no; NA: No meta-analysis. Item 1: Research question and whether the inclusion criteria include elements of PICO; Item 2: Whether to report that the methodology of the system evaluation study has been determined prior to implementation, and whether to report inconsistencies with the plan; Item 3: Whether the author explains the reasons for selecting the type of study design included in the systematic evaluation. Item 4: Whether the author uses a comprehensive literature search strategy; Item5: Whether two persons independently completed literature screening; Item 6: Whether data extraction is completed by two persons independently; Item7: Whether a list of excluded references is provided and the reasons for exclusion; Item 8: Whether the author’s description of the basic features of the included study is detailed ;Item 9: Whether the author uses appropriate tools to assess the risk of bias in the included literature; Item 10: Whether the author reports on the sources of funding for studies included in the systematic evaluation; Item 11: If the meta-analysis is carried out, whether the author USES appropriate statistical methods to merge the results for analysis; Item 12: If a meta-analysis is conducted, whether the author considers the potential impact of the bias risk of included studies on meta-analysis or other evidence integration; Item 13:Whether the author considers the risk of bias in the included study when interpreting or discussing the results of the system evaluation; Item 14: Whether the author gives a satisfactory explanation or discussion of heterogeneity in the results of systematic evaluation; Item 15: If quantitative synthesis is performed, whether publication bias has been adequately investigated and its possible influence on the results discussed; Item 16: Whether the author reports any potential conflicts of interest, including any funding received to conduct a systematic review.
